# Hierarchically structured porous materials: synthesis strategies and applications in energy storage

**DOI:** 10.1093/nsr/nwaa183

**Published:** 2020-08-24

**Authors:** Liang Wu, Yu Li, Zhengyi Fu, Bao-Lian Su

**Affiliations:** State Key Laboratory of Advanced Technology for Materials Synthesis and Processing, Wuhan University of Technology, Wuhan 430070, China; State Key Laboratory of Advanced Technology for Materials Synthesis and Processing, Wuhan University of Technology, Wuhan 430070, China; State Key Laboratory of Advanced Technology for Materials Synthesis and Processing, Wuhan University of Technology, Wuhan 430070, China; State Key Laboratory of Advanced Technology for Materials Synthesis and Processing, Wuhan University of Technology, Wuhan 430070, China; Laboratory of Inorganic Materials Chemistry (CMI), University of Namur, Namur B-5000, Belgium

**Keywords:** hierarchically structured porous materials, synthesis strategies, energy storage, porous parameters, geometric configuration

## Abstract

To address the growing energy demands of sustainable development, it is crucial to develop new materials that can improve the efficiency of energy storage systems. Hierarchically structured porous materials have shown their great potential for energy storage applications owing to their large accessible space, high surface area, low density, excellent accommodation capability with volume and thermal variation, variable chemical compositions and well controlled and interconnected hierarchical porosity at different length scales. Porous hierarchy benefits electron and ion transport, and mass diffusion and exchange. The electrochemical behavior of hierarchically structured porous materials varies with different pore parameters. Understanding their relationship can lead to the defined and accurate design of highly efficient hierarchically structured porous materials to enhance further their energy storage performance. In this review, we take the characteristic parameters of the hierarchical pores as the survey object to summarize the recent progress on hierarchically structured porous materials for energy storage. This is the first of this kind exclusively to survey the performance of hierarchically structured porous materials from different porous characteristics. For those who are not familiar with hierarchically structured porous materials, a series of very significant synthesis strategies of hierarchically structured porous materials are firstly and briefly reviewed. This will be beneficial for those who want to quickly obtain useful reference information about the synthesis strategies of new hierarchically structured porous materials to improve their performance in energy storage. The effect of different organizational, structural and geometric parameters of porous hierarchy on their electrochemical behavior is then deeply discussed. We outline the existing problems and development challenges of hierarchically structured porous materials that need to be addressed in renewable energy applications. We hope that this review can stimulate strong intuition into the design and application of new hierarchically structured porous materials in energy storage and other fields.

## INTRODUCTION

The highly evolving world economy has resulted in a significant increase in global energy requirement, leading to rising concerns of energy shortage and global warming problems for society. As a result, the development of highly efficient, clean and renewable energy sources such as wind, solar and ocean energy has become increasingly important and urgent [1,2]. However, the intermittence is one of the serious drawbacks of these renewable energy sources. One solution to the challenge of these renewable intermittent energy sources is investing in energy storage [3,4]. Recently, a lot of effort has been devoted to the research and development of various rechargeable and reversible energy storage devices such as lithium-ion batteries (LIBs) [5–7], sodium-ion batteries (SIBs) [8–10], lithium-sulfur (Li-S) batteries [11–13] and supercapacitors [14–16]. The results demonstrate that the achievement of electrochemical energy storage devices with both high energy and power densities urgently requires the design and preparation of advanced high-performance electrode materials [17–19].

According to the definition from the International Union of Pure and Applied Chemistry (IUPAC), porous materials are classified into three categories based on different pore sizes: microporous (<2 nm), mesoporous (2–50 nm) or macroporous (>50 nm) materials. Hierarchically structured porous materials exhibit a porous hierarchy in which the porosity and structure span multiple length scales from micro- to meso- and macropores. These unique structures have attracted much attention because of their diversity and good performance, and are largely used as an important family of functional materials in catalysis [20], photocatalysis [21], adsorption [22], separation [23], energy conversion and storage [24,25], sensing [26] to biomedicine [27,28] in recent years. These materials are generally characterized by multiple levels of porosity, the pore regularity at each level, high interconnection between each level of porosity, large accessible space, high surface area, low density and excellent volume change accommodation, which facilitates the transport of electron/ion and the diffusion of mass, showing great importance in energy storage technology [29–31]. As far as we know, the energy density, rate capacity and cycling life are several practical issues that are critical for reversible energy storage devices [32]. A hierarchically porous structure with highly vascularized and interconnected porosity at different length scales can reduce the ion diffusion pathway to enhance the depth of the electrochemical reaction [33], which makes the capacity of the electrode material fully play out and increases the energy density. It is also beneficial to the charge transfer, which can speed up the quick reversible de-intercalation of ions and improve the rate performance [34]. Moreover, it can enhance the structural stability of the electrode material for increasing the cycle life of energy storage devices, because the large porous space and the interconnection of pores at different length scales can accommodate not only the volume variation [35] but also heat absorption and dispersion [36] during cycling.

For decades, plenty of methods and technologies have been utilized and developed to prepare hierarchically structured porous materials, including soft templating methods and hard templating methods [37–39]. Besides, the self-formation method based on a spontaneous phenomenon, referring to the chemistry of organic metal alkoxides and alkylmetals, has also been deeply studied and successfully applied to the synthesis of a large variety of single metal oxides, binary oxides, ternary metal oxides, composites, silicoaluminates, and aluminophosphates with hierarchically porous architectures [37,38,40–44]. These methods can produce hierarchically structured porous materials with different number scale levels, sizes, shapes and orientations of pores. As mentioned above, since hierarchically structured porous materials can provide an efficient solution to the practical problems of energy storage, such as capacity loss, poor rate capability, volume expansion and limited cycle life, encountered in commercial application of reversible batteries and supercapacitors, their synthesis and energy storage applications have been reported by a series of remarkable reviews [28–31,37,38,45,46]. However, most of the reviews have focused on the progress of hierarchically structured porous materials in the field of energy storage, either from the chemical families of materials or from the application directions; there is no report summarizing the relationship between hierarchically structured porous materials and electrochemical properties based on pore parameters control. The pore parameters are of primary importance in the performance improvement of energy storage devices. The relationship between pore parameters and their electrochemical behavior in energy storage is thus essential.

From a structural and morphological point of view, hierarchically structured porous materials with vascularized branch systems have rich and diverse architecture due to the combination of different organizational, structural and geometric parameters of pores at different length scales. The number of scale levels in hierarchically structured porous materials is the first parameter to consider, which may be two-stage or three-stage distributions of pore sizes at a single scale level such as bimodal mesoporous, or multiple levels of pore sizes spanning two or three length scales such as micro-meso, meso-macro, micro-macro, or even micro-meso-macro levels. Secondly, it is known that there are larger pores and smaller pores in hierarchically structured porous materials, thus the respective sizes and ratios of the big pore and small pore is another important design parameter. In addition, pore shape, being also a key factor, can be columnar with the same upper and lower pore diameters, or a funnel-like pore with different upper and lower pore diameters. Moreover, these pores can be ordered or disordered. Even the orientation of their channels can be either centrally divergent or have parallel and vertical alignment. Therefore, the discussion on the application of hierarchically structured porous materials in energy storage based on pore parameters control is very meaningful, and important for the scientific and accurate design of hierarchically structured porous materials and the understanding of the relationship between structure and properties. ‘Materials-Properties-By Design’ is the concept for future development. Is it possible to establish materials design principles to achieve predictive and optimized functions for energy storage applications?

The present review aims at illustrating the benefits of hierarchically structured porous materials in energy storage and will be divided into four sections. After the introduction, in the second section, a very brief presentation on the synthesis of different hierarchically structured porous materials with various porosities at different length scales, different morphologies and different chemical composition will be given. In the third section, we will take the characteristic parameters of the hierarchical pores as the survey object to summarize the recent progress on hierarchically structured porous materials for energy storage applications. We will first discuss the hierarchically structured porous materials with different numbers of scale levels applied in the field of energy storage. For each scale level, the effect of their pore sizes on electrochemical performance will then be illustrated. We highlight the key developments in the quantitative matching of pore sizes range from micro- to meso- and macro-scale for energy storage. Next, we discuss hierarchically structured porous materials with different shapes and with either ordered or irregular porosity accompanied by comments on the orientation effect of the pore channels, because both are useful in energy applications. A conclusion and prospect pointing out the future research direction of hierarchically structured porous materials in the field of energy storage will be given in the last section. This is the first review summarizing the relationship between the organizational, structural and geometric parameters of pores at different length scales in materials and their electrochemical properties to try to set up some design and synthesis rules and guidance to achieve predictive and optimized functions in energy storage applications that can be extended to other fields.

## SYNTHESIS OF HIERARCHICALLY STRUCTURED POROUS MATERIALS

**Figure 1.**
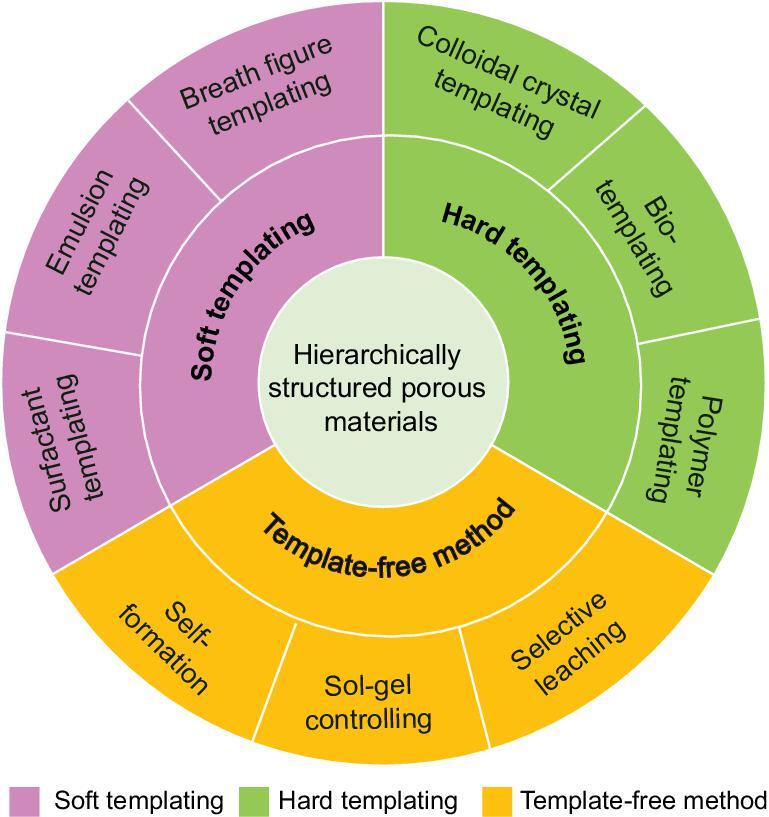
Illustration of various technologies for the synthesis of hierarchically structured porous materials: soft templating (surfactant templating, emulsion templating and breath figure templating), hard templating (colloidal crystal templating, bio-templating and polymer templating), and template-free method (self-formation, sol-gel controlling and selective leaching).

A wide range of chemical methods [47–53], physical-chemical methods [54–57] and chemically related engineering techniques [58–60] can be used to prepare hierarchically structured porous materials. According to whether the template is used and the type of template agents used, these methods are divided into soft templating method, hard templating method and template-free method. Each type of synthesis technology contains a series of exact synthesis routes. All these three synthesis technologies with precise synthesis routes are summarized in Fig. 1. The templating methods are the most commonly used strategies for the synthesis of materials with porous hierarchy. The hierarchically structured porous materials prepared by templating methods (including soft templating methods and hard templating methods) have tunable pore sizes and designable pore shapes. However, these methods involve a post template-removal process, which needs complicated procedures and requires more resources and energy. Template-free methods, which mainly relate to the spontaneous formation phenomenon and self-assembly process, are much simpler methods, but they are difficult to control and it is difficult to predict the size and shape of the pores due to the spontaneous formation character of the method. In the following parts, the synthesis of different hierarchically structured porous materials will be discussed on the basis of soft templating, hard templating and template-free technologies (Fig. 1).

### Soft templating

The soft templates generally refer to the molecular systems which have variable organization structures and limited domain ability in a certain space scope. In this part, we will introduce three major soft templating methods: surfactant templating, emulsion templating and breath figure templating.

#### Surfactant templating

**Figure 2.**
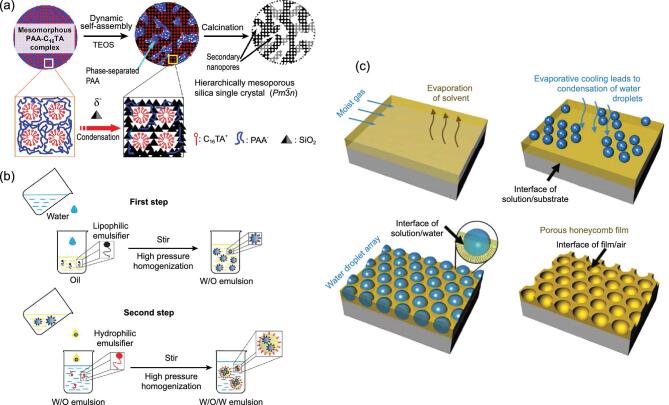
(a) Schematic illustration of the polyelectrolyte-assisted surfactant templating method for hierarchially porous silica (reproduced with permission [66], Copyright 2020, American Chemical Society); (b) schematic demonstration of a two-step fabrication process of the W/O/W emulsions (reproduced with permission [70], Copyright 2017, Royal Society of Chemistry); (c) schematic presentation of the formation of honeycomb films by the breath figure strategy (reproduced with permission [80], Copyright 2014, Royal Society of Chemistry).

Surfactant templating is a kind of method to synthesize porous materials by using multi molecular aggregates (micelles, liquid crystals, vesicles, etc.) as the structure-directing agents that are assembled by surfactant molecules. This method allows the creation of mesoporous materials with highly ordered channels and uniform pore size. In this sense, the combination of two kinds of surfactant molecules with different molecular sizes can produce hierarchically structured porous materials with dual mesoporous distribution [61]. Furthermore, hierarchically trimodal porous architectures were prepared by introducing tertiary macroscopical scale templates into double surfactant systems [62–64]. For example, Sel *et al.* produced hierarchically trimodal macro-meso-mesoporous materials by using polymer spheres as macro-template, block copolymers with large molecular weight and ionic liquids with small molecular weight as dual surfactant templates [65]. Recently, a surfactant-based dynamic template method—polyelectrolyte-surfactant mesomorphous complex templating method—has attracted much attention for its potential applications in the preparation of hierarchically structured porous materials [66]. In the synthesis process, the dynamic self-assembly induced by *in situ* phase separation mechanism plays a key role (Fig. 2a). By simply adjusting the types of surfactant and precursors, this method can fabricate hierarchically structured porous materials with different morphologies and chemical compositions, showing the excellent universality of the method.

The surfactant templating method has the advantage of controllable pore size and adjustable pore shape; it can synthesize a variety of hierarchically structured porous materials, including silicon dioxides, metals and metal oxides [48]. Moreover, as surfactant molecules are generally organic molecules, their *in situ* carbonization can enhance the conductivity of the materials without destroying the porous hierarchy, making this method an important tool in the field of energy storage.

#### Emulsion templating

Emulsion templating is based on the properties of emulsion, through the solidification or polymerization of emulsion in continuous phase to form good porosity in materials. It is a simple and relatively mild preparation method, which can be used to prepare hierarchically structured porous materials containing mesopores or macropores, because the size and distribution of emulsion particles can be controlled in the range of 10–1000 nm. There are three basic emulsion systems that are water-in-oil emulsion (W/O), oil-in-water emulsion (O/W) and supercritical CO_2_ emulsion (C/W). On this basis, more complex emulsion systems are designed. For example, Cooper *et al.* synthesized monodisperse porous polymer beads by sedimentation polymerization of oil-in-water-in-oil (O/W/O) emulsion [67]. Further, a series of hierarchically porous inorganic materials such as TiO_2_, Al_2_O_3_, ZrO_2_ and SiO_2_ are also prepared using the obtained porous polymer beads as templates [68,69]. Similarly, Wang *et al.* reported a water-in-oil-in-water (W/O/W) emulsion through a two-step emulsification method (Fig. 2b) [70]. These complex double emulsions are particularly suitable for the protection of bioactive compounds and widely used for medical applications.

Microemulsion and high internal phase emulsion are two common technologies for emulsion templating [71–73]. They have widely been used to prepare porous polymers, porous inorganic materials and porous inorganic-organic composites [74,75]. Although the emulsion template method has the advantages of strong adaptability and convenient implementation, there are still many problems to be solved in synthesizing hierarchically structured porous materials, such as the stability of the templated emulsion and the influence of template removal on material structure.

#### Breath figure templating

The breath figure (BF) method is named after a phenomenon whereby water vapor condenses on cold substrates to form ordered hexagonally arranged water droplets [76]. In this method, water droplets are used as templates; no special equipment is needed. The experimental conditions are mild and the operation procedure is simple, so it is one of the most effective methods for large-area fabrication of two-dimensional ordered porous structures [77–79]. Figure 2c shows the mechanism for the formation of the porous honeycomb films [80]. The interface of solution/water and other two interfaces including solution/substrate interface and film/air interface play an important role during the BF process, which can determine the structures of the films. Using this approach, star, block and graft copolymers as well as conjugated polymers or even inorganic materials with rigid segments can be used to prepare honeycomb-like ordered porous membranes with pore sizes ranging from several hundreds of nanometers to several hundreds of micrometers. Furthermore, modified breath figure techniques such as coupling with nanobuilding blocks allow for the synthesis of hierarchically macroporous membranes with mesoporous walls [81]. The experimental process of breath figure templating is non-isothermal and non-equilibrium. There are many variables affecting the structure in the experiment, including ambient temperature and humidity, air flow rate and solution concentration. In actual operations, this method is mostly based on empirical knowledge, so it is hard to obtain the expected structures by adjusting the experimental conditions. However, it is believed that with the further accumulation and summary of experimental data, the mechanism of breath figure templating will be more accurately and comprehensively understood.

### Hard templating

Hard templates are usually materials with relatively rigid structures to guide the growth of materials by limiting space. Compared with the soft templating approaches, the hard templating methods are more effective in precisely controlling the pore sizes due to their strong spatial confinement effect, but the hard templates are more difficult to remove. In this part, we will introduce three main hard templating technologies: colloidal crystal templating, bio-templating and polymer templating.

#### Colloidal crystal templating

**Figure 3.**
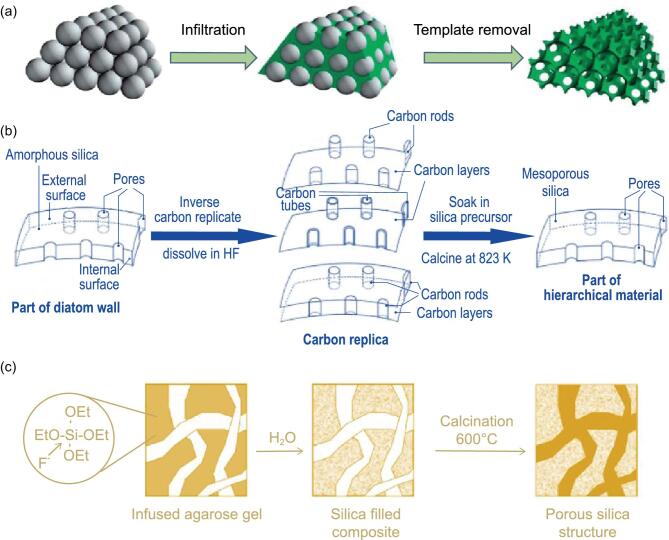
(a) Schematic representation of the preparation process of colloidal crystal templating method (reproduced with permission [82], Copyright 2013, Royal Society of Chemistry); (b) schematic demonstration of the synthesis of hierarchical meso/macroporous silica using diatom as bio-template (reproduced with permission [96], Copyright 2006, Wiley-VCH); (c) schematic illustration of the synthesis protocol for porous silica structure using agarose gel as hard polymer template (reproduced with permission [102], Copyright 2011, American Chemical Society).

Colloidal crystals are formed by self-assembly of monodisperse microspheres with three-dimensional (3D) ordered opal structure. The 3D ordered macroporous (3DOM) structure prepared by colloidal crystal templating method is the replication of this opal structure. The pores are periodically arranged, and their size can be easily adjusted by tuning the dimensions of the microspheres in the templates. This templating route comprises three basic steps as shown in Fig. 3a [82]: (i) the formation of a uniformly colloidal crystal template through various chemical processes; (ii) the infiltration of a fluid precursor; and (iii) the removal of the template to obtain a well-ordered porous replica. The secondary porosity such as mesopores can be obtained by combining the colloidal crystal template with surfactant templating through a commonly used sol-gel approach [83,84]. Therefore, the colloidal crystal templating method can be developed to synthesize hierarchically structured porous materials represented by 3DOM with mesoporous walls. The infiltration of precursors is a key step in the preparation of hierarchically 3DOM/mesoporous materials. A variety of techniques such as atomic layer deposition (ALD), chemical vapor deposition (CVD) and electrochemical deposition are often utilized for infiltration process [85,86]. The long-range ordered structure in hierarchically structured porous materials using colloidal crystals as templates makes these materials have many unique and potential applications, such as various optical signal processors with adjustable optical properties, and high performance electrochemical energy storage materials and devices with high mass transfer efficiency.

#### Bio-templating

The bio-templating method uses the natural biomass as template, through special treatment, retaining the structure of biological heritage to obtain porous materials with precise structure [87–95]. As a structural factory, there are many natural templates with special morphology and structure in nature. In particular, after billions of years of evolution and development, a rich variety of biological organisms have evolved to a natural hierarchical porous structure, which cannot be synthesized even by sophisticated modern technology, providing an ideal replication platform for preparing complex hierarchically structured porous materials [89]. The inherent hierarchically porous structure of biological materials endows them with many characteristics such as large specific surface area, light weight and high permeability. Meanwhile, the chemical composition and special microstructure of the organism can provide a stable and controllable environment for the synthesis of materials. Therefore, selecting suitable natural biological structures as templates can produce high-performance materials for practical industrial applications. For example, Cai *et al.* [96] synthesized the meso/macroporous hierarchical porous silica using diatoms as direct hard bio-templates (Fig. 3b). During the preparation process, techniques like infiltration and coating are utilized to immerse the templates for complete replication of whole hierarchical levels of biological materials [97–99]. The hierarchically structured porous materials prepared by bio-templating method often have abundant surface functional groups and easily controllable pore structures, these functional materials have an attractive application prospect in the fields of energy, catalysis, sensors and biomedicine.

#### Polymer templating

Porous polymeric materials with macroporous or mesoporous structure, such as polymer foams, polymer fibers and polymer gels, can be used as templates to prepare hierarchically structured porous materials [100,101]. Because of the characteristic advantages of the porous polymers, such as various preparation methods, adjustable pore size, and controllable surface modification and so on, when used as a template it can provide a platform where chemical reactions can take place, as well as a scaffold for infiltration of nanoparticles. Figure 3c illustrates the synthesis protocol for the polymer guided fabrication of porous silica [102]. Firstly, the precursor solution is injected into the porous polymer gel to form silica filled composite. Through the removal of polymer template, the porous silica retaining the structural properties of the initial template with pore size ranging from tens to 100 nanometers is then generated. Sol-gel chemistry and microfluidic technology are commonly used to prepare macroporous polymers [103,104]. The utilization of macroporous polymer templates combining with surfactant templates can result in hierarchically structured macro-mesoporous materials. Besides, additional microporous structures can also be created by introducing the zeolite crystals.

### Template-free methods

From the above presentation, it is clear that the templating methods including soft templating methods and hard templating methods are very effective ways for the synthesis of hierarchically structured porous materials. However, drawbacks related to high cost and tedious preparation procedure have hampered mass production for large scale applications. Ideally, template-free methods for fabrication of hierarchically structured porous materials with a wide scale of pore sizes and variable shapes would be preferred. In this part, three simpler and commonly used template-free synthetic strategies, including self-formation, sol-gel controlling and selective leaching, will be surveyed.

#### Self-formation

**Figure 4.**
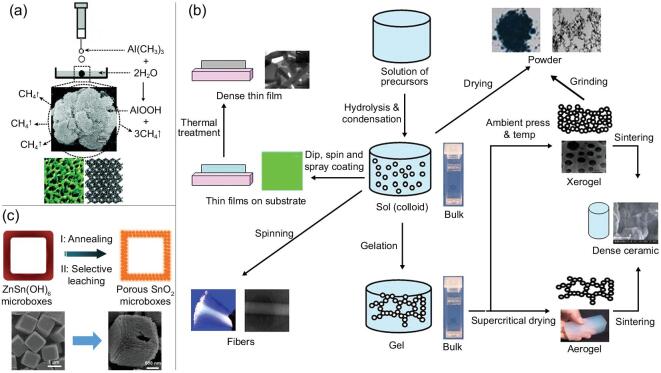
(a) The self-formation procedure for preparing hierarchically structured porous alumina foams on the basis of a spontaneous phenomenon (reproduced with permission [40], Copyright 2010, American Chemical Society); (b) schematic diagram of the synthesis process of the sol-gel method for the preparation of porous materials with various shapes (reproduced with permission [109], Copyright 2008, IOP Publishing Ltd.); (c) schematic representation of the formation of porous hollow SnO_2_ microboxes through selective leaching approach and their corresponding SEM images (reproduced with permission [119], Copyright 2014, Royal Society of Chemistry).

The self-formation method, based on a spontaneous phenomenon, referring to the chemistry of organic metal alkoxides and alkylmetals, has been deeply studied and successfully applied to the synthesis of hierarchically structured porous architectures of a large family of materials [37–44,105–107]. The concept of ‘poregene’ can be used to describe the mechanism of this process. As a poregene, the water and alcohol molecules quickly released during the rapid hydrolysis and condensation of metal alkoxides play a key factor, leading to the formation of parallel macroporous channels from the inside to the outside in the materials. The very uniform mesoporous structure formed by the aggregation of particles of tens of nanometers in the material constitutes the walls of macropores. In the final materials, the particles of tens of nanometers themselves also have a microporous structure by the aggregation of smaller nanoparticles. On the basis of such a self-formation procedure, a wide range of hierarchically structured porous materials (single metal oxides such as Y_2_O_3_, ZrO_2_, Ta_2_O_5_, Al_2_O_3_ and Nb_2_O_5_; mixed metal oxides such as Y_2_O_3_/ZrO_2_, TiO_2_/ZrO_2_, Al_2_O_3_/SiO_2_ and aluminosilicates, aluminophosphates, etc.) have been successfully synthesized [37,38,40–44,105–107]. In addition to metal alkoxides, alkylmetals can also be used as starting reactants for the spontaneous self-formation procedure. Li *et al.* [40], for example, reported a simple one-pot template-free self-formation route for synthesis of hierarchically structured meso/macroporous alumina foams by using trimethylaluminum as a generator of poregene (Fig. 4a). The unique advantage of the self-formation process is the high purity of final materials obtained with any post-treatment. The preparation procedure is very simple and quite mild at room temperature and ambient conditions without the addition of acid or base to control the reaction. The drawback is the cost of precursors such as metal alkoxides and metal alkyls. The spontaneously formed hierarchically structured porous materials with multi-scale interconnected micro-meso-macropores possess highly efficient diffusion and exchange properties and have shown great potential in nanotechnology, energy conversion and cell therapy.

#### Sol-gel controlling

The sol-gel method based on sol-gel chemistry is an important strategy in synthesizing inorganic porous materials under mild conditions [108]. The raw materials were firstly dispersed in the solvent. The active monomers are formed through hydrolysis, alcoholysis or coordination reactions, leading to the formation of a sol. The sol then transforms gradually to gel with a certain spatial structure. Finally, the required solid materials are prepared by drying and heat treatment [109]. Monoliths, fibers, films and powder materials with various appearances can be prepared (Fig. 4b). In the sol-gel process, the structures of the final products are closely related to the gel structure, thus the properties of the sol. Therefore, the quality of the sol is very important. Factors that affect the quality of the sol, such as the amount of water, pH value, concentration and solvent effect, should be strictly controlled [110–113]. The sol-gel method facilitates the arrangement of inorganic species on the molecular scale, which is very important in controlling the physical and chemical properties of materials. However, in the process of preparation, the capillary shrinkage force caused by the gelation will lead to the shrinkage of the pores and the decrease of the porosity. Therefore, in the actual operation process, the sol-gel method often needs to be combined with template methods. This is due to the fact that a combination of appropriate organic additives can reduce the liquid surface tension, so that the pore structures of the final products can be improved. This greatly expands the applications of the sol-gel method to the synthesis of hierarchically structured porous materials.

#### Selective leaching

The immiscible two-phase composites with the sacrificial and desired phases can be used as precursors for selective leaching to prepare a variety of hierarchically structured porous materials, including metal oxides and metals. The general process of this template-free route is described as follow: firstly, an intimately mixed composite is obtained by some physical or chemical techniques such as evaporation, sintering and decomposition of mixing precipitates; then, the sacrificial phase is dissolved through chemical reactions to leave a porous skeleton of the desired phase [114–116]. Chemical etching and electrochemical etching are the two most common selective leaching methods, in which chemical etching can be done by acid-washing or selective reaction of gases [117–119]. For example, Zhang *et al.* [119] reported the preparation of porous hollow SnO_2_ microboxes through selective leaching of Zn(II) species from an Sn-based mixed composite by a simple acid-washing process (Fig. 4c). The uniformity of pore structure is directly affected by the homogeneity of phase distribution. Metal-organic frameworks (MOFs) is a new family of porous materials with regular pore structures formed by the connection of metal ions and organic ligands [120–123]. Recently, high-orientation etching of MOF particles can be realized to fabricate hollow MOFs through precise engraving by selective leaching [124]. It can greatly improve the chemical kinetics of these materials, which provides new ideas to prepare the hierarchical MOFs with more complex morphologies in the future. Such selective leaching method enables the pore size and porous hierarchy to be easily tuned by adjusting the starting phase ratio, particle and available etching mediums. However, the waste of sacrifice phase consumption is also a problem that cannot be ignored.

**Figure 5.**
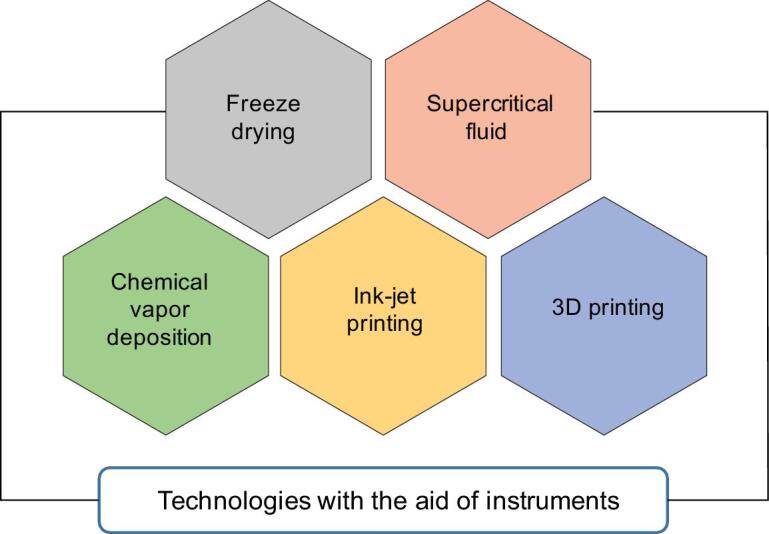
Examples of some engineering techniques that rely on specialized equipment for the synthesis of hierarchically structured porous materials.

In addition to the above methods, some engineering techniques that require specialized equipment can also be used for the preparation of hierarchically structured porous materials (Fig. 5), such as freeze drying [125,126], supercritical fluid [127,128], chemical vapor deposition [129], ink-jet printing [58] and 3D printing [130], etc. In order to obtain the ideal hierarchical porous structure, these technical means often have high requirements on operating experience and process parameters.

All the synthesis strategies described here, with different hierarchically structured porous materials with defined morphologies, various chemical compositions and diverse porous hierarchy, are generally summarized in Table 1. For one desired hierarchically structured porous material, different synthesis methods can be chosen and can be combined. This section of review can be helpful for those who are not familiar with the synthesis of hierarchically structured porous materials. In the following section, some innovative and very particular synthesis methods leading to specific hierarchically structured porous materials for defined energy storage application will also be introduced.

**Table 1.** Strategies employed for the synthesis of hierarchically structured porous materials and corresponding morphologies and compositions.

Synthesis methods	Porosities	Morphologies	Compositions	Ref.
Surfactant templating	Micro–mesopores; meso–macropores; dual mesopores; dual meso–macropores	Powders; films; spheres; monoliths	Oxides; carbon	[61–66]
Emulsion templating	Meso–macropores; multiple macropores	Powders; spheres; films; foams; monoliths	Oxides; polymers; carbon; metals; composites	[67–75]
Polymer templating	Micro–macropores; meso–macropores	Powders; fibers; tubes; spheres; films; membranes; foams; monoliths	Oxides; carbon; metals; composites	[76–81]
Colloidal crystal templating	Micro–macropores; meso–macropores; dual meso–macropores; dual macropores	Powders; films; monoliths	Oxides; polymers; carbon; metals; composites	[82–86]
Bio-templating	Micro–macropores; meso–macropores	Powders; fibers; tubes; films; membranes; monoliths	Oxides; carbon; composites	[89,96–99]
Breath figures templating	Meso–macropores; dual/multiple macropores	Powders; films	Oxides; polymers; composites	[100–104]
Self-formation	Meso–macropores; micro–meso–macropores	Powders; spheres; monoliths	Oxides; composites	[105–107]
Sol–gel controlling	Micro–macropores; dual mesopores; micro–mesopores; micro–meso–macropores; meso–macropores	Powders; foams; fibers; spheres; films; membranes; monoliths	Oxides; polymers; carbon; metals; composites	[108–113]
Selective leaching	Meso–macropores; multiple macropores	Powders; monoliths	Oxides; composites	[114–124]

## HIERARCHICALLY STRUCTURED POROUS MATERIALS FOR ENERGY STORAGE

**Figure 6.**
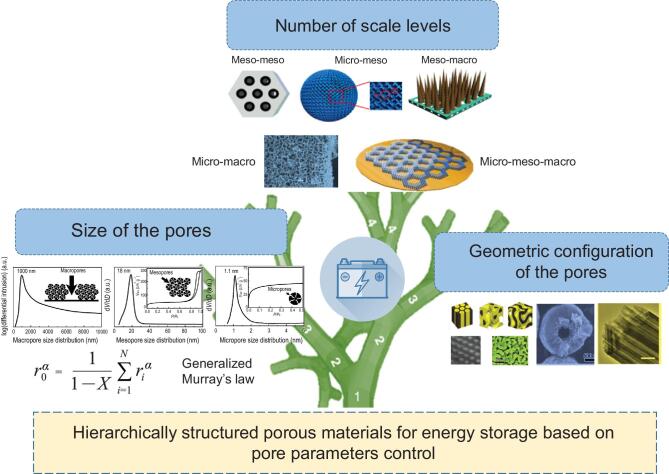
Schematic representaton of hierarchically structured porous materials for energy storage based on pore parameters control.

The organizational, structural and geometric parameters of pores can significantly affect the electrochemical properties of the hierarchically structured porous materials. In this section, we will discuss the application of hierarchically structured porous materials in energy storage based on pore parameter control. These parameters include number of the scale levels, pore size and geometric configuration (see Fig. 6). Discussing the correlation between organizational, structural and geometric parameters of pores at different length scales and electrochemical performance is of great significance to the design and application of hierarchically structured porous materials to realize the concept of ‘Materials-Properties-By Design’. It also helps to establish the design guidance to target predictive and optimized functions in energy storage application. Table 2 summarizes the detailed pore parameters, chemical compositions, synthesis methods and performance of the selected hierarchically structured porous materials for various applications of energy storage.

**Table 2.** Summary of the representative hierarchically structured porous materials for energy storage.

Pore parameters	Materials	Methods	Applications	Performance	Ref.
Bimodal mesopores	Mn_2_O_3_	Precipitation-decomposition	Li-ion battery	845 mAh g^−1^ after 50 cycles at 100 mAh g^−1^, 600 mAh g^−1^ after 100 cycles at 1000 mAh g^−1^	[132]
Bimodal mesopores	TiO_2_	Solvothermal process	Li-ion battery	188 mAh g^−1^ after 100 cycles at 1 C	[133]
Bimodal mesopores	LiFePO_4_/C/G	Solid-state reaction	Li-ion battery	103 mAh g^−1^ after 500 cycles at 10 C	[134]
Bimodal mesopores	Li_x_MnO_2_	Electrodeposition	Li-ion battery	260 mAh g^−1^ after 50 cycles at 0.1 C	[135]
Bimodal mesopores	CMK-5 carbon	Replication	Li-S battery	419 mAh g^−1^ after 100 cycles at 0.3 C	[137]
Bimodal mesopores	CNT/Graphene −Li_2_S	Solvothermal reaction	Li-S battery	958 mAh g^−1^ after 300 cycles at 0.2 C	[138]
Bimodal mesopores	CNT/graphene–sulfur	Solvothermal reaction	Li-S battery	975 mAh g^−1^ after 200 cycles at 0.2 C	[139]
Bimodal mesopores	Carbon nanofibers	Two-step casting process	Li-S battery	550 mAh g^−1^ after 150 cycles at 1 C	[140]
Bimodal mesopores	CoO@BMC	Wet-chemistry impregnation approach	Li-O_2_ battery	Initial capacity of 4033 mAh g^−1^	[142]
Bimodal mesopores	TiN/Carbon	Molecular cooperative assembly	Li-O_2_ battery	Onset potential of OER is at 3.0 V	[143]
Bimodal mesopores	A-Co@CMK-3-D	Templating	Zn–air battery	A half-wave potential of 0.835 V vs. RHE	[144]
Bimodal mesopores	NiO	Hydrothermal method	Supercapacitor	112 F g^−1^ at 2 A g^−1^, 3.2% decrease after 500 cycles	[145]
Micro–mesopores	Carbon nanocages	Co-precipitation	Li-S battery	780 mAh g^−1^ after 60 cycles at 0.2 C	[158]
Micro–mesopores	Carbon	Self-assembly	Supercapacitor	160 F g^−1^ in ionic liquid electrolyte and 210 F g^−1^ in 1 M H_2_SO_4_ aqueous electrolyte at 0.25 A g^−1^	[159]
Micro–mesopores	Carbon	Nanopore lithography	Supercapacitor	246 F g^−1^ in 1 M H_2_SO_4_ electrolyte at 2 mV s^−1^	[160]
Micro–mesopores	MOFs/graphene/ CNT	Microfluidic method	Supercapacitor	Volumetric capacitance of 472 F cm^−3^ at 0.1 A cm^−3^, 94.2% of retention after 10 000 cycles	[161]
Micro–mesopores	N-doped carbon	Sol-gel process and CVD	Supercapacitor	790 F g^−1^ in 0.5 M H_2_SO_4_ electrolyte at 1 A g^−1^	[162]
Micro–mesopores	N-doped carbon	Supramolecular assembly	Supercapacitor	537 F g^−1^ in 0.5 M H_2_SO_4_ electrolyte at 0.5 A g^−1^, 98.8% of retention after 10 000 cycles at 5 A g^−1^	[163]
Micro–mesopores	PANI/carbon	CVD	Supercapacitor	136 F g^−1^ in 1 M H_2_SO_4_ electrolyte at 10 mV s^−1^, 100 F g^−1^ after 10 000 cycles at 20 mV s^−1^ with 90% of retention	[164]
Micro–mesopores	Carbon nanofibers	Electrospinning method	Li-S battery	700 mAh g^−1^ after 300 cycles at 0.5 C	[165]
Micro–mesopores	N-doped carbon	Bio-templating	Li-S battery	971 mAh g^−1^ after 100 cycles at 0.1 C, 578 mAh g^−1^ after 500 cycles at 1 C	[166]
Micro–mesopores	Graphene and carbon	Thermolysis process	Li-S battery	916 mAh g^−1^ after 100 cycles at 0.2 C, 550 mAh g^−1^ after 450 cycles at 2 C	[167]
Micro–mesopores	CeO_2_/carbon	Etching	Li-S battery	1066 mAh g^−1^ after 200 cycles at 0.2 C, 721 mAh g^−1^ after 1000 cycles at 2 C	[168]
Micro–mesopores	Graphene	KOH activation process	Li-S battery	502 mAh g^−1^ after 100 cycles at 0.15 C	[169]
Micro–mesopores	Carbon	Bio-templating	Li-S battery	586 mAh g^−1^ after 200 cycles at 1 C	[170]
Micro–mesopores	Carbon	Selective leaching	Li-Se battery	420 mAh g^−1^ after 150 cycles at 0.2 C	[171]
Micro–mesopores	N-doped carbon	Pyrolysis of MOFs	Li-Se battery	555 mAh g^−1^ after 150 cycles at 0.2 C, 462 mAh g^−1^ after 200 cycles at 0.5 C	[173]
Micro–mesopores	N-doped carbon	Pyrolysis and etching	Na-Se battery	612 mAh g^−1^ after 200 cycles at 0.2 C	[174]
Micro–mesopores	Carbon/P composite	Pyrolysis and etching	Na-ion battery	1675 mAh g^−1^ after 100 cycles at 0.5 A g^−1^, 1269 mAh g^−1^ after 1000 cycles at 2 A g^−1^	[177]
Micro–mesopores	Carbon-silica composite	Self-assembly and etching	Li-ion battery	611 mAh g^−1^ after 200 cycles at 0.2 A g^−1^, 313 mAh g^−1^ after 1500 cycles at 3 A g^−1^	[178]
Micro–mesopores	Sulfur-doped carbon	Sol-gel method with co-assembly	Na-ion battery	443 mAh g^−1^ after 50 cycles at 50 mA g^−1^, 238 mAh g^−1^ after 600 cycles at 1 A g^−1^	[179]
Meso–macropores	TiO_2_	Colloidal crystal templating	Li-ion battery	144 mAh g^−1^ after 200 cycles at 1 C, 106 mAh g^−1^ after 200 cycles at 4 C	[181]
Meso–macropores	TiO_2_	Biomolecular self-assembly	Li-ion battery	186 mAh g^−1^ after 200 cycles at 1 C, 161, 145, 127 and 97 mAh g^−1^ after 1000 cycles at 2, 5, 10 and 20 C	[182]
Meso–macropores	TiO_2_/C	Sol-gel method and carbonization	Li-ion battery	188 mAh g^−1^ after 200 cycles at 0.5 C	[183]
Meso–macropores	LiFePO_4_/C	Microwave assisted solvothermal process	Li-ion battery	166 mAh g^−1^ after 200 cycles at 0.2 C, 87.2% of retention after 1000 cycles at 2 C	[184]
Meso–macropores	NiO	Self-assembly	Li-ion battery	710 mAh g^−1^ after 1000 cycles at 1 A g^−1^	[185]
Meso–macropores	ZnCo_2_O_4_/MnO_2_	Hydrothermal method	Supercapacitor	2339 F g^−1^ at 1 A g^−1^, retention of 95.9% after 3000 cycles at 2 A g^−1^ and 94.5% after 8000 cycles at 10 A g^−1^	[186]
Meso–macropores	CuCo_2_O_4_/NiO	Hydrothermal method	Supercapacitor	2219 F g^−1^ at 1 A g^−1^, retention of 95.3% after 10 000 cycles at 20 A g^−1^	[187]
Meso–macropores	Nitrogen-enriched carbon	Co-electrospinning followed by pyrolysis and etching	Supercapacitor	242 F g^−1^ at 0.2 A g^−1^, retention of 99% after 5000 cycles at 1 A g^−1^	[188]
Meso–macropores	TiO_2_	Hydrolysis and pyrolysis of MOFs	Li-ion battery	145 mAh g^−1^ after 200 cycles at 5 C	[193]
Micro–macropores	Carbonaceous monoliths	Hard templating	Li-ion battery	200 mAh g^−1^ after 50 cycles at 0.1 C	[195]
Micro–macropores	Carbon	Bio-templating	Li-S battery	760 mAh g^−1^ after 200 cycles at 0.2 C	[196]
Micro–macropores	Carbon	Bio-templating	Li-S battery	877 mAh g^−1^ after 50 cycles at 0.1 C	[197]
Micro–meso–macropores	Carbon	Bio-templating	Li-O_2_ battery	18 114 mAh g^−1^ with a discharge plateau of 2.80 V at 100 mA g^−1^	[198]
Micro–meso–macropores	Carbon	Bio-templating	Supercapacitor/Li-ion battery	474 F g^−1^ with capacitance retention of 95.6% over 20 000 cycles (for supercapacitor); 266 mAh g^−1^ after 800 cycles at 1 A g^−1^ (for Li-ion battery)	[199]
Micro–meso–macropores	Carbon	Bio-templating and etching	Supercapacitor	326 and F g^−1^ in 1.0-V and 1.8-V	[200]
Micro–meso–macropores	Carbon	Salt template-assisted chemical activation	Supercapacitor	260 F g^−1^ in 1 M H_2_SO_4_ electrolyte at 0.1 A g^−1^, retention of 90% after 10 000 cycles at 5 A g^−1^	[201]
Micro–meso–macropores	N/P co-doped carbon	Polymerization and co-assembly	Supercapacitor	200 F g^−1^ at 0.5 A g^−1^ and 132 F g^−1^ at 20 A g^−1^ in 6 M KOH electrolyte	[202]
Micro–meso–macropores	Spheres-in-tube carbon	Confined assembly followed by carbonization and etching	Supercapacitor	235 F g^−1^ at 0.2 A g^−1^ and 156 F g^−1^ at 20 A g^−1^ in 6 M KOH electrolyte	[203]
Micro–meso–macropores	FeCo-N-C	MOFs and polystyrene sphere (PS) templating	Li-O_2_ battery	18 750 mAh g^−1^ at 0.1 A g^−1^, 7900 mAh g^−1^ at 0.5 A g^−1^	[204]
Micro–meso–macropores	N-doped carbon	CaCO_3_ spheres template-induced self-activation	Electric double layer capacitors	6.2 and 14.4 Wh kg^−1^ in 6 M KOH and 5 M LiTFSI electrolyte, respectively	[205]
Micro–meso–macropores	Graphene	Templating and CVD	Li-S battery	434 mAh g^−1^ after 150 cycles at 0.5 C	[206]
Micro–meso–macropores	TiO_2_@carbon-rGO	Electrospinning and etching	Li-ion battery	250.1 mAh g^−1^ at 0.1 A g^−1^ and remains a capacity of 117.9 mAh g^−1^ at 2 A g^−1^	[207]
Defined pore sizes obeyed Murray's law	ZnO	Self-assembly	Li-ion battery	1260 mAh g^−1^ after 5000 cycles at 2.5 A g^−1^	[217]
Single gyroidal shape	Carbon	Co-assembly of block copolymers	Li-S battery	380 mAh g^−1^ after 100 cycles at 0.1 C	[218]
Double gyroidal shape	Carbon	Co-assembly of block copolymers	Li-S battery	400 mAh g^−1^ after 100 cycles at 0.1 C	[218]
Hexagonally packed cylindrical shape	Carbon	Co-assembly of block copolymers	Li-S battery	380 mAh g^−1^ after 100 cycles at 0.1 C	[218]
Cage-like shape	Graphitic-carbon	Self-assembly	Li-S battery	950 mAh g^−1^ after 100 cycles at 0.5 C, 729 mAh g^−1^ after 400 cycles at 1 C	[219]
Micro-mesopores with ordered mesopores	Carbon	Self-assembly	Supercapacitor	160 F g^−1^ in ionic liquid electrolyte and 210 F g^−1^ in 1 M H_2_SO_4_ aqueous electrolyte at 0.25 A g^−1^	[159]
Meso-macropores with ordered macropores	TiO_2_	Colloidal crystal templating	Li-ion battery	144 mAh g^−1^ after 200 cycles at 1 C, 106 mAh g^−1^ after 200 cycles at 4 C	[181]
Bimodal mesopores with disordered pores	Mn_2_O_3_	Precipitation-decomposition	Li-ion battery	845 mAh g^−1^ after 50 cycles at 100 mAh g^−1^, 600 mAh g^−1^ after 100 cycles at 1000 mAh g^−1^	[132]
Meso–macropores with disordered pores	TiO_2_	Biomolecular self-assembly	Li-ion battery	186 mAh g^−1^ after 200 cycles at 1 C, 161, 145, 127 and 97 mAh g^−1^ after 1000 cycles at 2, 5, 10 and 20 C	[182]
Radially oriented pores	TiO_2_	Solvothermal alcoholysis route	Li-ion battery	216 mAh g^−1^ after 100 cycles at 1 C, 112 mAh g^−1^ after 100 cycles at 10 C	[222]
Parallel pores	Carbon	Electrospinning	Li-S battery	950 mAh g^−1^ after 200 cycles at 0.2 C	[224]

### Number of scale levels

#### Hierarchical porosity within a single scale level

In general, hierarchically structured porous materials describe a family of materials containing porous hierarchy with interconnected pores on multiple length scales from micro-, meso- to macropores, such as bimodal combination of micro-meso, meso-macro and micro-macro or trimodal combination of micro-meso-macro, or even presenting two-stage and multi-stage pore diameter distribution within a single scale level. The larger the diameter of pores, the smaller the influence of their size difference, so the design of hierarchical character at only one single macro-scale level is of little significance. Bimodal microporous and bimodal mesoporous are thus the two most common examples for hierarchy design within a single scale level. However, only a few zeolite materials have been found to have a unique bimodal microporous structure [131], which are not used for electrochemical devices due to their electrical insulating behavior. Therefore, in this part, we will mainly focus on hierarchical bimodal mesoporous structure for energy storage application. Compared with single pore size mesoporous materials, hierarchical bimodal mesoporous materials with a proper combination of characteristics of both small and large mesopore size can provide higher accessibility for ions transport, better permeability for electrolyte infiltration and larger space for volume expansion. Therefore, many efforts have been made to design and synthesize this kind of hierarchically bimodal mesoporous structure for various energy storage devices, including LIBs [132–136], Li-S batteries [137–141], Li-O_2_ batteries [142,143], zinc-air batteries [144] and supercapacitors [145,146].

**Figure 7.**
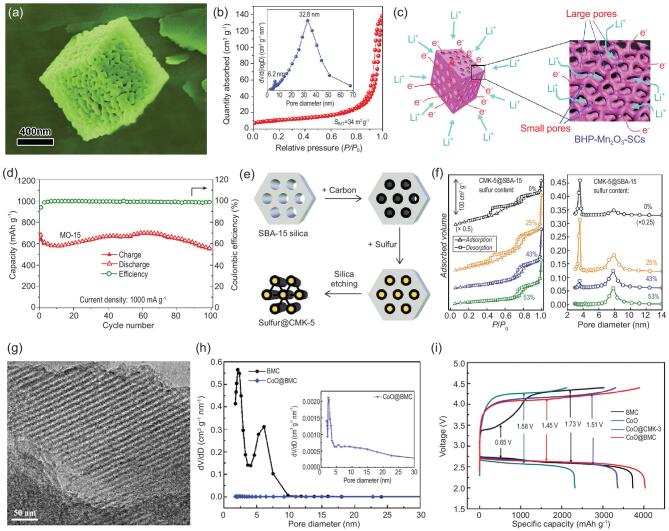
(a) SEM image, (b) N_2_ adsorption-desorption isotherms, the inset in (b) is the pore size distribution, (c) illustration of the mechanism of fast electron transportation in the 3D bicontinuous hierarchically bimodal mesoporous Mn_2_O_3_ single crystals (BHP-Mn_2_O_3_-SCs) and (d) cycling performance of BHP-Mn_2_O_3_-SCs (reproduced with permission [132], Copyright 2015, Macmillan Publishers Ltd.); (e) schematic diagram of synthesis route for the incorporation of sulfur in bimodal mesoporous CMK-5 carbon, and (f) N_2_ adsorption-desorption isotherms and BJH pore size distribution plots of the bimodal mesoporous CMK-5@SBA-15 host materials with different sulfur contents (reproduced with permission [137], Copyright 2018, American Chemical Society); (g) TEM image of BMC, (h) pore size distribution of BMC and CoO@BMC, and (i) voltage profile of the first cycle for BMC, CoO, CoO@CMK-3 and CoO@BMC under a current density of 0.075 mA cm^−2^ (reproduced with permission [142], Copyright 2016, Elsevier Ltd.).

Through a simple precipitation-decomposition method, the hierarchically porous Mn_2_O_3_ single crystals with interconnected bimodal mesoporous architecture (BHP-Mn_2_O_3_-SCs) has been prepared and used as anode material for LIBs [132]. These BHP-Mn_2_O_3_-SCs have a homogeneous parallelepiped structure with a very developed porosity after decomposition of MnCO_3_ precipitate by heat treatment (Fig. 7a). The pore size distribution plot by N_2_ adsorption-desorption measurement displays that they have a bimodal mesoporosity with large mesopores mainly centered at 32.8 nm and small mesopores narrowly centered at 6.2 nm (Fig. 7b). As shown in Fig. 7c, the interconnected continuous large mesopores and small mesopores can work together to facilitate the transfer of electrons and ions. In addition, owing to their specific hierarchical bimodal mesoporosity, the volume change of electrode materials is well accommodated by large mesopores because of their abundant void space. The chemical kinetics are greatly elevated by small mesopores due to their large specific surface areas. Meanwhile, their combination is very favorable for electron/ion transport at the interface between electrode and electrolyte, leading to excellent cycling performance. The specific discharge capacity of the BHP-Mn_2_O_3_-SCs can be maintained as high as 845 mAh g^−1^ after 50 cycles at a current density of 100 mA g^−1^, which is superior to most of the results from Mn_2_O_3_-based bulk solids and nanostructures. Even when the current density increases to 1 A g^−1^, the BHP-Mn_2_O_3_-SCs still show a high specific capacity of ∼600 mAh g^−1^ with ∼100% coulombic efficiency (Fig. 7d).

In addition to the anode material, the cathode material is also an important active component of the lithium-ion batteries. Generally, the energy density of a rechargeable battery is determined by the specific capacity and operating voltage of the cathode. This is because the charge and discharge capacity of cathode materials are much lower than those of anode materials; the energy density of the batteries depends thus on that of cathode materials. The structural design to improve the performance of positive electrode materials has thus been the main focus of research in recent years [147–149]. Using a solid-state reaction method assisted by ball milling technology, Wang *et al.* prepared carbon-coated LiFePO_4_/graphene nanocompsites (LFP@C/G) with well-defined hierarchical bimodal mesoporosity [134]. The Barret-Jouner-Halenda (BJH) pore size distribution curve of LFP@C/G shows bimodal mesoporous distributions, centered at ∼3.8 and ∼9.5 nm, respectively. This unique ‘sheets-in-pellets’ bimodal mesoporous structure exhibits an outstanding rate capability with a high initial discharge capacity of 164 and 147 mAh g^−1^ at 0.1 C and 1 C and good cycling stability with a high capacity retention of 92% after 500 cycles at 10 C. The superior properties are attributed to its abundant mesopores, which are beneficial for electrolyte ions transport and diffusion to improve the cycling and rate performance of olivine-type LiFePO_4_ electrode. A similar finding has been found in porous Li_x_MnO_2_ cathode [135]. Its bimodal mesoporous structure includes small primary mesopores within a narrow range of sizes (diameter range: 2.0–4.0 nm, with a distribution centered at ∼2.5 nm) and larger secondary mesopores with a diameter ranging from 4.0 to 30.0 nm centered at 12.0 nm. The hierarchical bimodal mesoporous structure has a larger pore volume, which is able to offer the buffering spaces against the volume variation during the Li^+^ insertion/extraction process and a high interfacial contact area between the electrolyte and active material, resulting in excellent electrochemical performance with high specific discharge capacity of 283.0, 240.0, 191.0, 161.0 and 113.0 mAh g^−1^ at 0.1, 0.2, 1, 2 and 5 C, respectively.

In terms of cost and specific energies, Li-S batteries have an advantage over LIBs, making them greatly desirable for high-performance energy storage systems [11–13,150–154]. So far, a variety of porous carbon matrices have been developed as composite cathodes of Li-S batteries for sulfur loading [155–157]. In order to increase the sulfur loading and suppress the shuttle effect, the structure of porous carbon materials needs to be carefully designed. Recently, bimodal mesoporous CMK-5 carbon [137] has been prepared as the conducting host for active cathodes of sulfur. Their small mesopores confine polysulfides, reducing the shuttle effect, while their larger mesopores facilitate the electrochemical kinetics (Fig. 7e and f). This CMK-5 carbon with 53 wt% sulfur delivered an initial discharge capacity as high as 1107 mAh g^−1^ because of its hierarchical bimodal mesoporosity. Similar improvements in performance were obtained from bimodal mesoporous carbon foam and bimodal mesoporous carbon nanofibers by introducing hierarchical porous networks in the carbon matrix [138,141].

Moreover, the confinement of various metal oxides [142], metal nitrides [143], metal atoms [144] and perovskites into bimodal mesoporous carbon can be an effective strategy in producing stable cathode electrocatalysts for Li-O_2_ batteries and zinc-air batteries. For example, CoO has been confined into two types of carbon materials with different porous structures [142], which are bimodal mesoporous carbon (CoO@BMC) with hierarchical porosity and CMK-3 carbon (CoO@CMK-3) with single mesoporosity (Fig. 7g and h). Due to the larger specific surface areas offered by the unique hierarchical bimodal mesoporous structure, the CoO@BMC nanocomposite can efficiently catalyze the decomposition of Li_2_O_2_ and even some carbonates generated in the discharging process, presenting a much higher initial capacity over 4000 mAh g^−1^ compared with pure BMC, pure CoO and CoO@CMK-3 (3359 mAh g^−1^) (Fig. 7i). Lyu *et al.* produced atomic Co catalysts stably implanted in defective bimodal mesoporous CMK-3 carbon (A-Co@CMK-3-D) for zinc-air batteries [144]. Its hierarchically porous structure offers more bimodal mesoporous channels with a specific surface area as high as 1447 m^2^ g^−1^ guaranteeing the facile infiltration of the electrolyte and the diffusion of oxygen bubbles for durable oxygen reduction reaction (ORR), leading to efficient electrochemical performance on ORR in an alkaline electrolyte with a half-wave potential (0.835 V vs. reversible hydrogen electrode (RHE)), which is comparable to that of Pt/C (0.839 V vs. RHE). In addition to carbon-based materials, bimodal mesoporous NiO [145] and silica [146] were also successfully synthesized and used as active electrodes for high performance supercapacitors due to the synergy effect of the different mesopore sizes on the capacitive properties.

#### Hierarchical porosity spanning two scale levels

Pores with different size range at different scale levels have different functions. Micropores provide the shape and size selectivity for guest ions and molecules, mesopores offer a large pore volume and a high special surface area for enhancing the interactions between host and guest, and macropores can considerably improve the diffusion to, and the accessibility of, the active sites for guest ions and molecules. Although the hierarchy design within a single scale level represented by bimodal mesoporous structure can improve the electrochemical performance of the materials to some extent, the intrinsic properties of their large mesopores and small mesopores are similar due to the fact that they are at the same scale range. Therefore, it is more desirable to fabricate porous materials with hierarchical porosity spanning two scale levels, which can combine the advantages of two different types of pores.

##### Hierarchically micro-mesoporous level.

Due to the extremely slow mass transfer through micropores, it is a promising way to introduce a mesoporous network into microporous materials by using the principle of hierarchical structure design, which can eliminate the transport limitation [33] and thus enhance their performance of energy storage. The synergistic effect of bimodal pore systems at different length scales is particularly beneficial for supercapacitors, because the size of the micropores is close to the diameter of ions resulting in optimum energy storage performance, and the mesopores can be used as quick ion channels to promote the ions diffusion into the small micropores, thus achieving high rate and power performance. Moreover, hierarchical micro-mesoporous structures are also desirable in lithium-sulfur batteries [158], as the unique porous structure can help to suppress the escape of lithium polysulfides by micropores as well as facilitate the transport of Li^+^ ions and increase sulfur loading via mesopores.

**Figure 8.**
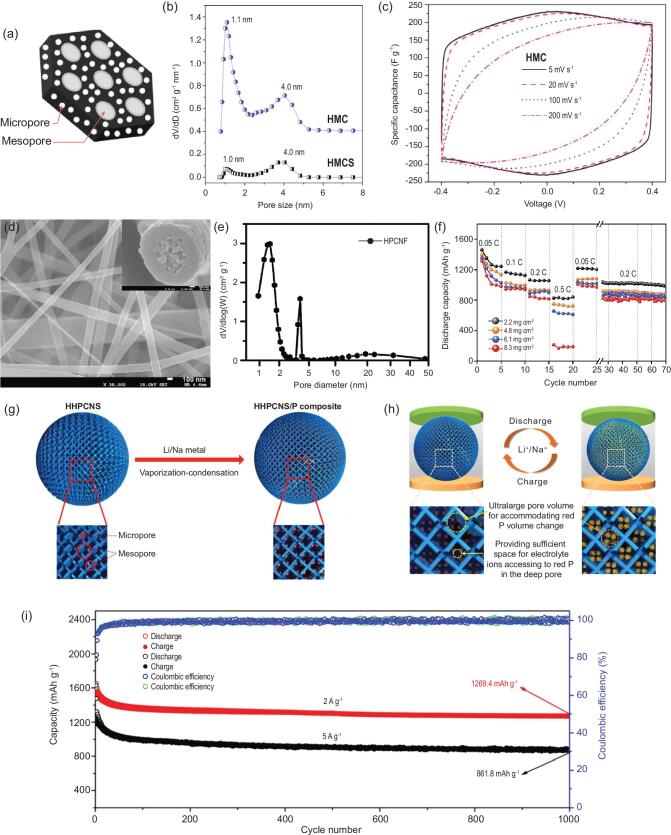
(a) Schematic diagram, (b) pore size distribution and (c) CV curves under different scan rates of the fabricated hierarchically micro-/mesoporous carbons fabricated by co-assembly method (reproduced with permission [159], Copyright 2016, Elsevier Ltd.); (d) SEM image and cross-section (inset) of the hierarchically structured porous carbon nanofiber (HPCNF) prepared by a facile electrospinning method, (e) pore size distribution of HPCNF, (f) rate performance of Li-S cells with S/HPCNF at different sulfur loading (reproduced with permission [165], Copyright 2018, Elsevier Ltd.); (g) schematic illustration of the synthesis process for the honeycomb-like hierarchical micro-mesoporous carbon nanospheres and red P composite (HHPCNSs/P), (h) schematic illustration of the advantages of the HHPCNSs as the host of red P, and (i) long-term cyclic stability of the HHPCNSs/P composite electrode for 1000 cycles at 2 and 5 A g^−1^ for SIBs, respectively (reproduced with permission [177], Copyright 2019, American Chemical Society).

Through a co-assembly method based on the chemistry of polyhedral oligosilsesquioxanes (POSS), the hierarchically micro-/mesoporous carbons with quite uniform micropores and highly ordered mesopores have been synthesized [159] (Fig. 8a), and have a high specific surface area of more than 2000 m^2^ g^−1^ with pore volume up to 1.19 cm^3^ g^−1^. The micropores are narrowly centered at 1.1 nm and the high-quality honeycomb-like hexagonal mesopores centered at 4 nm (Fig. 8b). Benefiting from the abundant microporosity and mesoporosity, the materials can provide more charge storage interfaces and shorter ion diffusion paths, displaying excellent rate capability with 97% capacitance retention in 1 M H_2_SO_4_ at a wide range current density from 0.25 to 10 A g^−1^ (Fig. 8c). MOFs derived carbon materials tend to be mostly microporous; combining them with carbon materials with rich mesoporous structure such as graphene oxide (GO) nanosheets and carbon nanotubes (CNTs) is an effective strategy in constructing hierarchically micro-/mesoporous materials for high performance supercapacitors [160,161]. In such structures, the large mesopores will serve as highways for the electrolyte to penetrate and evenly cover the electrode material, and contact quickly with the micropores, enhancing the power density and rate performance. It is worth noting that the capacitance of such hierarchically micro-mesoporous material is not always satisfying owing to their relatively low surface area compared to highly microporous material. In addition to keeping the micro-mesoporous structure, material modification such as heteroatom doping [162,163] and surface coating of conductive polymers [164], which can generate additional pseudocapacitance for enhancing the performance, can improve the capacitance performance.

The important role of micro-mesoporous hierarchy is also widely revealed in lithium-sulfur batteries [165–170] and lithium-selenium batteries [171,172]. Zhao *et al.* [165] fabricated root-like porous carbon nanofibers with bimodal size distributions of meso- and micropores narrowly centered at 4.1 and 1.8 nm, respectively (Fig. 8d and e). The multi-scaled pores act as an efficient host for the confinement of large amounts of sulfur, and accommodate the associated volume change and reduce the escape of polysulfides during electrochemical cycling. These finely fabricated root-like micro-mesoporous carbon nanofibers allowed the highest loading amount of sulfur so far reported, up to 12.1 mg cm^−2^, and exhibited a high sulfur utilization of >80% with a real capacity reaching to 11.3 mAh cm^−2^. Moreover, by rationally tuning the ratio of micropores to mesopores, an optimum capability among Li-S battery systems can be attained. Because mesopores are more important for a high cyclic retention, materials with a higher proportion of mesoporous volume thus displayed a capacity up to 1032 mAh g^−1^ and a high retention with decay rate of 0.055% per cycle at a current density of 0.1 C (Fig. 8f). In addition to the electrode material, the modification on separator is also an effective method in restraining the shuttle effect of polysulfide. Recently, Shao *et al.* prepared a modified separator coated with a micro-/mesoporous nitrogen-doped carbon layer (N-MIMEC) for Li-S batteries [166], exhibiting excellent electrochemical behavior of a high initial discharge capacity of 1301 mAh g^−1^ with 75% capacity retention after 100 cycles at 0.1 C. Because a N-MIMEC coated separator can effectively suppress the shuttle effect of lithium polysulfide (LiPS) during a long-cycle deeply charging/discharging process, the battery exhibited a high capacity of 578 mAh g^−1^ after 500 cycles with a ultralow decay rate of only 0.029% per cycle even at a high current density of 1 C. Li-Se systems and Li-S systems have similar electrochemical processes, which also face the challenges of polyselenide dissolution during the cell operation. The success of the hierarchical porosity concept in lithium-sulfur batteries highlights the advantage of using a micro-/mesoporous hierarchy to overcome the problems troubling to lithium-selenium batteries [173].

Recently, research on sodium-ion batteries is booming because of their low cost due to the abundant sodium resource. The radius of sodium-ion (1.02 Å) is 55% larger than that of lithium-ion (0.76 Å); the graphite anode commonly applied in commercial LIBs is thus not appropriate for SIBs. Carbon materials with microporosity have been reported to be promising anodes for sodium ion batteries due to their relatively good electrical conductivity, chemical stability and low cost. However, the capacity of these electrode materials needs to be

improved, since their microporous structures cannot be fully utilized leading to slow ion transportation. It has been suggested that hierarchically micro-/mesoporous structures can greatly enhance the electrochemical properties of carbonaceous materials in SIBs or Na-Se batteries [174–176], mainly due to the obtained abundant active sites and transport channels and to the fact that micro-mesoporous structures can store more sodium ions, provide fast diffusion channels for ion transfer, and buffer the volume change during the repeated sodium-ion storage process. The hierarchical characteristic advantage of micro-mesoporous structures can also be evidenced for loading active materials with high theoretical capacity such as phosphorus and silicon for sodium-ion batteries [177–179]. For example, porous carbon nanospheres with hierarchical porosity (HHPCNSs) of micro-mesopores and ultra-large pore volume of 3.258 cm^3^ g^−1^ have been synthesized and used for encapsulation of red phosphorus to prepare HHPCNSs/P composite by a facile vaporization-condensation method [177] (Fig. 8g and h). The obtained composite electrode delivers a capacity reaching up to 1674.8 mAh g^−1^ after 100 cycles at 0.5 A g^−1^ and an excellent long cycling stability with a low capacity decay rate of 0.02% per cycle after 1000 cycles at 5 A g^−1^ for SIBs (Fig. 8i). The unique honeycomb-like hierarchically micro-mesoporous nanostructure possessing ultra-large pore volume can not only increase electrode-electrolyte contact areas, but also accelerate Na^+^ migration to improve capacity and rate performance. There is also a large space to accommodate the huge volume variation of red P during charge-discharge process, which exhibits excellent electrochemical properties. As shown in Fig. 8h, the mesopores provide ultra-large pore volume for accommodating red P volume change, while the micropores offer abundant void space for electrolyte ions diffusion and accessibility to red P.

##### Hierarchically meso-macroporous level.

Generally, mesoporous materials having a pore structure of thin pore walls (<20 nm) and short channels (<100 nm), are very suitable for energy storage systems due to the shortened transport length of ions and electrons. However, mesoporous materials suffer from the limited diffusibility due to confined nano-channels. Incorporation of macropores in mesoporous materials can combine merits from both the mesoporous and macroporous structures. Compared with mesoporous materials with single-sized porosity, hierarchically meso-macroporous structures with interconnected channels have enhanced properties because of the increased mass transport through the material and the maintenance of a specific surface area on the level of fine pore systems [38,180], making them ideal candidates for energy storage [181–184].

**Figure 9.**
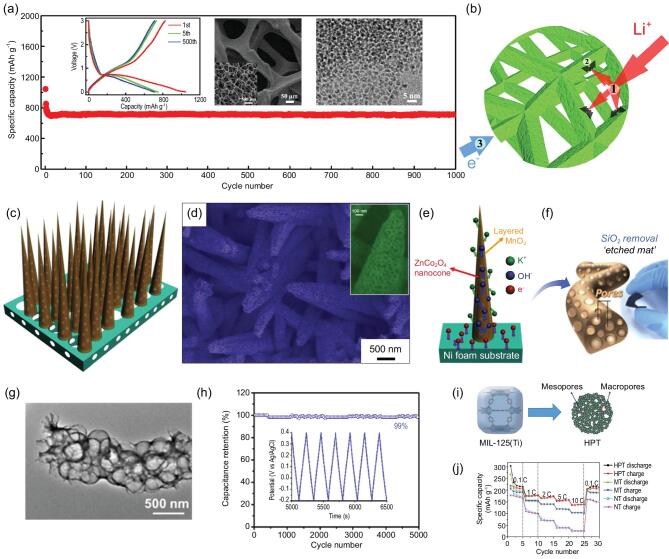
(a) SEM image, TEM image and cycling performance of the hierarchically meso-macroporous NiO/NF electrode, and (b) illustration of the Li-ion transfer in the electrolyte/electrode and electron conduction in the electrode (reproduced with permission [185], Copyright 2015, Elsevier Ltd.); (c) schematic diagram and (d) SEM image of the ZnCo_2_O_4_/MnO_2_ composite nanocone forests (NCFs) with a hierarchical core/shell structure, and (e) schematic representation of rechargeable supercapacitor based on ZnCo_2_O_4_/MnO_2_ NCFs on Ni foam (reproduced with permission [186], Copyright 2014, Elsevier Ltd.); (f) fabrication schematic presentation, (g) TEM image and (h) cycling performance of meso-macroporous nitrogen-enriched carbon fiber (reproduced with permission [188], Copyright 2016, Elsevier Ltd.); (i) schematic illustration of the formation mechanism and (j) rate performance of the hierarchically meso-/macroporous TiO_2_ (HPT) (reproduced with permission [193], Copyright 2015, Royal Society of Chemistry).

Nickel foam (NF) has a 3D interconnected macroporous network with good electrical conductivity. Growing mesoporous materials on nickel foam is very promising for the synthesis of hierarchically meso-macroporous materials [185–187]. Through the self-assembly of nanosized NiO on a macroporous conductive network of nickel foam, a binder-free hierarchically meso-macroporous electrode has been prepared, as anode for lithium-ion battery, showing supercapacitor-like electrochemical behavior and high performance [185] (Fig. 9a). The mesopores with a narrow mean size of 7 nm can favor the quick diffusion of ions and enhance the reaction kinetics, while the macropores of NF interconnecting with mesopores provide fast channels for the infiltration of active electrodes by electrolyte (Fig. 9b). Such hierarchically interconnected meso-macroporous electrode architecture has an electrolyte-filled conductive network, which facilitates rapid ion transfer at electrode/electrolyte interface and fast ion diffusion in solid phase. Taking advantage of this structure, the hierarchically meso-macroporous NiO/NF electrode displays high rate performance similar to that of supercapacitor while maintaining the high energy density of a lithium-ion battery during charge/discharge process. Remarkably, at a very high current density of 50 A g^−1^, it still delivers a high reversible capacity of more than 500 mAh g^−1^, showing superior cycling stability and excellent rate capability. Similarly, Qiu *et al.* prepared mesoporous nanocone forests of ZnCo_2_O_4_/MnO_2_ [186] (Fig. 9c–e) and nanotrees constructed by CuCo_2_O_4_ trunks and NiO branches on macroporous substrate of 3D nickel foam [187]. Due to the unique 3D hierarchical meso-macroporous networks, these two composite electrodes exhibit excellent performance as supercapacitors.

Metal oxides etching method is also a common approach for preparing macroporous materials as described in the second section [188–191]. Through co-electrospinning of polymer/SiO_2_ blended solution followed by pyrolysis and SiO_2_ removal processes (Fig. 9f), Fan *et al.* [188] synthesized hierarchically meso-macroporous nitrogen-enriched carbon fiber (NCF). Such NCF exhibits a thin carbon layer morphology and interconnected meso-macroporous nanostructure (Fig. 9g). Benefiting from the shortened ion diffusion path, it displays high pseudocapacitance (242 F g^−1^), fast rate-responses and stable cycling lifetime (99% of initial capacitance after 5000 cycles) coupled with purely capacitive behaviors (Fig. 9h).

Metal-organic frameworks formed by self-assembly of organic ligands and metal ions or clusters via coordination bonds are a new type of functional inorganic-organic hybrid material with high intramolecular porosity, large surface area and tunable pore structure, and can be used as templates to fabricate hierarchically meso-macroporous materials [192,193]. Xiu *et al.* reported a facile and mild approach to fabricate anatase TiO_2_ with hierarchically bimodal meso-macroporous structure via the hydrolysis of Ti-based MOF of MIL-125 followed by a heat treatment process in air [193] (Fig. 9i). The obtained material presented excellent rate capability (Fig. 9j) and good long-term cycling stability with a low capacity loss of only 6.5% at a high current density of 5 C after 200 cycles. This result can be attributed to its special hierarchically structured porous nanoarchitecture, which provides shorter diffusion lengths for both electron and Li^+^ transport and larger electrode-electrolyte contact areas.

##### Hierarchically micro-macroporous level.

**Figure 10.**
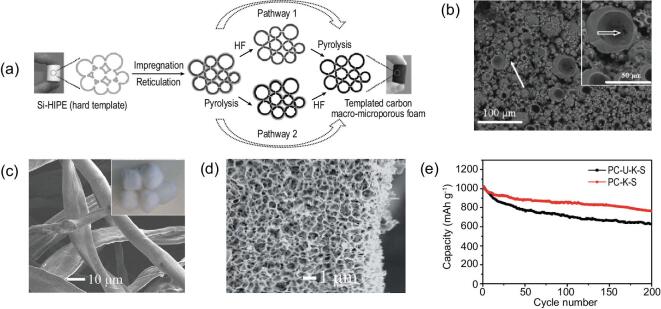
(a) Schematic illustration of the hard templating process for hierarchically micro-/macroporous carbonaceous monolith and (b) SEM micrograph of the resulting carbon material (reproduced with permission [195], Copyright 2009, Wiley-VCH); (c) SEM images of raw cotton, (d) SEM image of KOH treated hierarchically micro-/macroporous carbon (PC-K) from cotton and (e) cycling performance of the fabricated hierarchically porous carbon at 0.2 C for Li-S battery (reproduced with permission [196], Copyright 2014, Royal Society of Chemistry).

Microporous structure generally provides active reaction centers, whereas macroporous structure offers a fast pathway for mass transportation, making hierarchically micro-macroporous material a popular kind of bimodal porous structured material [194], for example, microporous zeolite materials containing additional macroporosity, which increases the transport efficiency of reactants, intermediates and products in the application of heterogeneous catalysis. This leads to a superior selectivity and activity as well as an increased stability of these catalysts compared to conventional systems [28]. Besides the catalysis application, the matching of these two porosities can also bring advantage for energy storage application. Recently, by using silica high-internal-phase emulsion (Si(HIPEs)) foams as hard exotemplating matrices, carbon monoliths with interconnected micro-macroporosity are prepared, showing a high specific surface area of ∼600 m^2^ g^−1^ [195] (Fig. 10a and b). The large macropores allow electrolyte to easily diffuse into the depths of the electrode material, facilitating the fast transport of conductive ions, while their abundant micropores contribute to a large specific surface area and a high reaction activity. As anode for lithium-ion batteries, it shows a good cyclability, in spite of initial irreversibility. At a current density of 0.1 C, a stable capacity of 200 mAh g^−1^ is obtained during the first 50 cycles.

The small pore size of the microporous materials has a strong physical confinement effect to reduce the shuttle effect of polysulfide, however, they suffer from an extremely slow mass transfer process. Hierarchically micro-macroporous materials can effectively suppress the shuttle effect while greatly enhancing the electrochemical properties of lithium-sulfur batteries. Wang *et al.* [196] prepared hierarchically micro-macroporous carbon materials with large specific surface areas from cotton (Fig. 10c and d). The macroporous structure of this carbon offers plenty of accessible channels which facilitates the sulfur loading into micropores and enhances fast transport of electrolyte and electrons/ions. Micropores in the structure trap polysulfides to suppress the shuttle effect during cycling. Therefore, the carbon/S composite electrode presents a high reversible capacity of 760 mAh g^−1^ after 200 cycles at 0.2 C as well as an excellent rate performance (Fig. 10e). Another activated hierarchically porous carbon has been obtained by means of chemical activation of waste mandarin peels with potassium carbonate [197]. The unique sponge-like micro-macroporous structure can be traced back to the original fibrous structure of the biomass sample. The material appeared to be very effective as a conducting component of the sulfur cathode, enabling the trapping of sulfur and intermediate polysulfides within the carbon network, thus contributing to a very high initial capacity and good cycling stability.

#### Hierarchical porosity spanning three scale levels

Given the short ion diffusion distances to the interior surfaces of macroporous materials, the low resistant pathways for the ions through the porous particles of mesoporous ones and the high electrochemical activity of microporous ones, hierarchically micro-meso-macroporous materials spanning three scale levels are regarded as being highly suited for electrochemical energy storage application, owing to their triple overlap advantages.

**Figure 11.**
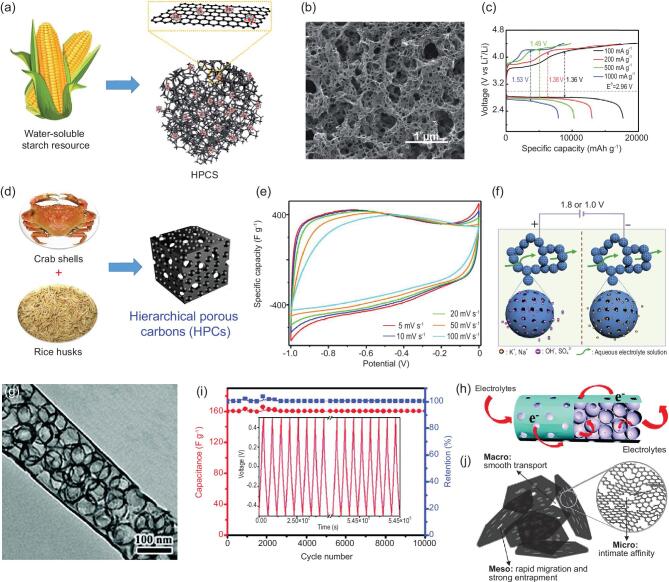
(a) Schematic illustration of the fabrication process of hierarchically porous carbon skeleton (HPCS) by using water-soluble starch as carbon source, (b) SEM image of HPCS and (c) rate capability of IrCo@HPCS cathodes (reproduced with permission [198], Copyright 2019, Royal Society of Chemistry); (d) schematic diagram of the fabrication process for hierarchically structured porous carbons (HPCs) derived from mixed biowastes of crab shells and rice husks and (e) rate dependent CV curves of the as-resulted porous carbons in three-electrode system (reproduced with permission [199], Copyright 2019, American Chemical Society); (f) scheme of the charge process of HPC-based aqueous symmetrical supercapacitor (reproduced with permission [200], Copyright 2017, Elsevier Ltd.); (g) TEM image of hollow carbon spheres encapsulated in one carbonaceous nanotube (HCSs@CT), (h) transport mechanism of electrons and ions and (i) cycling life test of HCSs@CT (reproduced with permission [203], Copyright 2018, Wiley-VCH); (j) illustration of the hierarchically micro-meso-macro porous graphene material used as the cathode scaffolds of Li-S battery (reproduced with permission [206], Copyright 2016, Wiley-VCH).

Recently, economical, green and abundant biomass precursors have been widely utilized as resources to fabricate hierarchically micro-meso-macroporous electrode materials, due to their natural and rich porous structure [198–201]. Shen *et al.* [198] prepared a hierarchically porous carbon skeleton (HPCS) by using water-soluble starch as carbon source (Fig. 11a and b). As a novel cathode nanostructure for Li-O_2_ batteries (LOBs), the air electrode consisting of HPCS can not only provide multiple interconnected channels (micro-, meso- and macroporous channels) for rapid electrolyte penetration and mass transfer (O_2_ and Li^+^) as well as abundant active sites for tri-phase interface reactions, but also offer sufficient storage space for insoluble discharge products. Thus, LOBs with HPCS catalysts exhibited high discharge capacities and good rate performance (Fig. 11c). Employing a KOH activation process to a bio-templating method, the hierarchically porous carbons (HPCs) were also fabricated, in which crab shells and rice husks were selected as carbon source [199] (Fig. 11d). The as-fabricated HPCs exhibit highly interconnected hierarchical micro-meso-macroporosity. This unique architecture gives them a large specific surface area, which facilitates electrode/ion transport and offers abundant active reaction sites. Using 6 M KOH as electrolyte, the resulting HPCs show superior performance for supercapacitors with a high capacitance (474 F g^−1^) and excellent cyclic stability (retention of 95.6% after 20 000 cycles) (Fig. 11e). Additionally, used as anode materials for LIBs, a high specific capacity and good cyclic stability are also obtained from HPCs. Similarly, Huang *et al.* [200] reported a hierarchically porous carbon prepared by using natural *Indicalamus* leaves and polytetrafluoroethylene as carbon precursor and silica as hard template, respectively. The size of micropores is mainly centered at ∼0.7 and 1.3 nm. Mesopores show a maximum pore size distribution peak at 34 nm, and macropores range from 50 to 100 nm. The unique 3D interconnected porous networks guarantee rapid ion transfer/diffusion and provide highly effective electrochemically accessible surface areas for formation of electric double layer (Fig. 11f). Benefiting from this valuable hierarchical porous structure, the obtained HPC demonstrates exceptional attractive electrochemical performances when used as the electrode of aqueous symmetrical supercapacitors.

Through carbonization of organic compounds and metal organic frameworks, it can be easy to produce hierarchically micro-/mesoporous materials. Combined with the hard templating method, a macroporous network can be introduced into the materials [202–205]. Zhang *et al.* designed a porous carbon microsphere with hollow cores and hierarchically micro-meso-macroporous shells through co-assembly of melamine-formaldehyde resin (MF) and colloidal SiO_2_ spheres followed by a two-step carbonization and template-removal process [202]. Due to the synergistic effect of the interconnected micro-, meso- and macropore structure, the obtained electrode materials could effectively improve the electro-active surface area and enhance the ion diffusion as well as electron transport. As ideal electrode materials for supercapacitors, they showed a high specific capacitance (200 F g^−1^ at 0.5 A g^−1^) as well as enhanced rate performance (132 F g^−1^ at 20 A g^−1^). Encapsulating hollow carbon spheres into one-dimensional nanotube (HCSs@CT), a novel spheres-in-tube hierarchical architecture has been constructed by Chen *et al.* [203] (Fig. 11g). In their design, the SiO_2_@ anodic aluminum oxide (AAO) dual template was prepared by assembling SiO_2_ nanoparticles in AAO channels used to coat polypyrrole (PPy) which was then carbonized and finally removed to form HCSs@CT. The pyrolysis process of PPy was studied, and the micro/mesopores with a size from 0.81 to 15 nm were obtained. In addition, meso/macropores from 20 to 100 nm were obtained mainly due to the removal of SiO_2_ templates. This interesting nanostructure combines the advantages of large specific surface area and good hierarchically micro-meso-macro porosity. Their synergy facilitates the diffusion of electrons and ions (Fig. 11h). Therefore, the resulting HCSs@CTs exhibit remarkable properties as active electrodes for supercapacitors (Fig. 11i). Chao *et al.* [204] prepared Fe/Co nanoparticles decorated 3D hierarchically micro-meso-macroporous carbon composites (M-FeCo-N-C-X) derived from FeCo bimetallic MOF as precursor and polystyrene microspheres (PS) as template. As cathode catalysts for Li-O_2_ batteries, the micropores in M-FeCo-N-C-X have strong capability in O_2_ capture and dictate the nucleation and early-stage deposition of Li_2_O_2_. The mesopores provide channels for the electrolyte penetration, and the macroporous structure promotes more accessible active sites. Thus, the obtained cathode exhibits a high initial capacity (18 750 mAh g^−1^@0.1 A g^−1^), good rate capability (7900 mAh g^−1^@0.5 A g^−1^) and a long cycling life of 192 cycles.

Besides hierarchically porous carbon spheres, hierarchically porous graphenes are also promising candidates for various energy storage systems [206,207]. Adopting porous CaO particles as both the catalyst and template for the CVD growth of graphene, a hierarchically porous graphene material was simply prepared [206]. The obtained material has hierarchical micro-meso-macroporosity with in-plane micropores, wrinkled mesopores and strutted macropores, thereby resulting in rapid mass transport and short ion diffusion distance due to the synergistic effect of pores at different length scales (Fig. 11j). As cathode material for Li-S battery, it delivers a reversible capacity of more than 650 mAh g^−1^ at a high current density of 5 C, which accounts for 74% of the capacity at 0.1 C, showing excellent rate capability. Also, this material displays high coulombic efficiency and exceptional cycling stability, it is thus proved to be a favorable host material for lithium-sulfur batteries.

### Size scale of the pores

For hierarchically structured porous materials, pore size is a very important parameter. The pore size affects the specific surface area and the efficiency of ion and electron transport, thus ultimately determining the electrochemical properties of the material. Especially for Li-S batteries, the pore size is of great significance in suppressing shuttle effect and increasing sulfur loading. In addition, the pore size, especially the size of micropores, plays an important role in improving the performance of supercapacitors. There are pores within different scale levels in hierarchically structured porous materials, so when considering the size of pores, we should not only look at the size of pores within a certain size range, but also consider the matching problem of the size of pores range from different scale levels. It is still a big challenge for materials to achieve high performance by accurately matching the aperture sizes of pores among different size ranges. Most of the researches on size matching of pores within different scale levels are still in the stage of qualitative description, while the research on quantitative matching of pore sizes range from micro to macro of hierarchically structured porous materials is just beginning.

#### Individual effects of pore size at different single length scales

**Figure 12.**
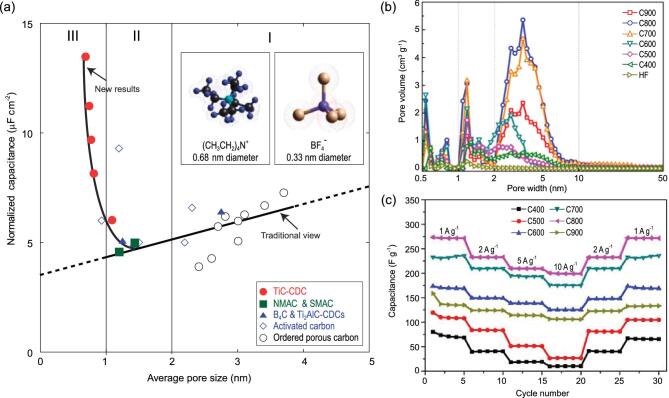
(a) Diagram of the relationship between the size of micropores and the capacitive performance (reproduced with permission [208], Copyright 2006, American Association for the Advancement of Science); (b) pore size distribution of hierarchically micro-mesoporous carbon materials prepared under different temperatures and (c) their rate capacitive performance at different current density (reproduced with permission [209], Copyright 2017, Elsevier Ltd.).

The pores in materials are the channels for mass transfer, providing the sites where the reaction takes place. Depending on pore size, the confinement effect could be variable. As one of the key parameters, the size of the pores significantly affects the materials’ properties. For hierarchically porous materials, the synergy effect between pores at different size-scale levels is crucial in influencing the performance. However, this requires a prerequisite of an understanding of the ‘size-performance relationship’ within a single scale. Generally, the micropore is the terminal of substance transport and the active center of reaction, and thus its size effect attracts large attention. With carbide-derived carbons as the object of research, Chmiola *et al.* investigated the effect of pore size of micropores on the capacitance of supercapacitors [208]. They prepared a series of micropores with different average sizes ranging from 0.6 to 2 nm. In order to eliminate the influence of a specific surface area, the capacitance is normalized. The results show that the capacitance increases with the decrease of micropore size when it is smaller than 1 nm due to the squeezed distortion of the solvated ions in the process of passing through narrow pores (Fig. 12a). These findings provide a new theoretical guidance to the pore structure design for porous carbon materials. Indeed, reducing the micropore size will appropriately be beneficial for the electronic devices seeking high energy density. In particular, micropores as reservoirs for active materials in Li-S and Li-Se batteries determine the phase morphology of sulfur or selenium and the atom numbers of their molecular chain, which will affect the electrochemical behavior of electrode materials. Meanwhile, by reducing the size of micropores, a better effect of inhibiting the shuttle of polysulfides will be achieved. In hierarchically micro-mesoporous materials, the outlets of micropores lead to mesopores. Therefore, the size of mesopores also has an important influence on the properties of materials. Hierarchically micro-mesoporous carbons with different mesoporous sizes were prepared by Yang *et al.* through tuning the synthesis temperature [209]. Compared with the materials prepared at 600°C, which have an average pore size of 2.2 nm, porous carbons prepared at 800°C have a larger mesoporous size of 3.2 nm (Fig. 12b) that is beneficial to the penetration of electrolyte and the migration of ions. This finally leads to better capacitive rate performance (Fig. 12c). In this sense, it is highly desirable to design materials with narrow micropores and relatively large mesopores for improving both energy and power properties. With similar mesopore size among different materials, the influence of mesoporous proportion becomes obvious; materials with a high ratio of mesopores exhibit better power characteristics due to their fast ion migration rate. In electrode materials, macropores have similar functions with mesopores, which also facilitate electrolyte infiltration and mass transfer, but relatively speaking, the change of macropore size has little effect on the properties of materials. Namely, in most of the research of hierarchically macroporous materials, not the size effect but the synergy effect is the main concern.

#### Hierarchy principle on quantitative matching pore sizes across multiple length scales

**Figure 13.**
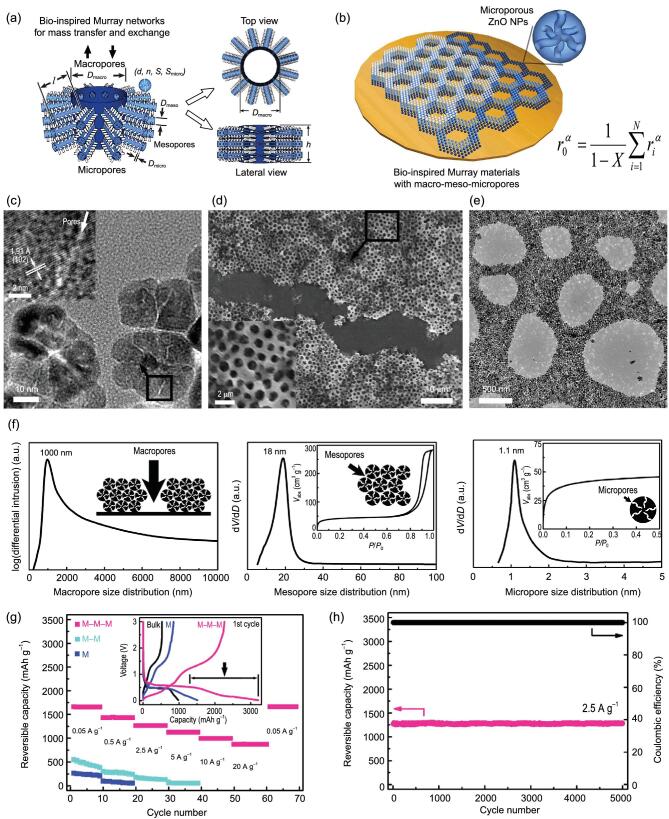
(a) Hierarchically structured porous network model abstracted from hierarchically macro-meso-microporous materials; (b) schematic diagram of the macro-meso-microporous ZnO Murray materials with defined pore size following the generalized Murray's law; (c) HRTEM image of microporous ZnO NPs, (d) SEM image, (e) TEM image and (f) pore size distribution curves for macropres, mesopores and micropores; (g) rate performance and (h) cycling performance of the fabricated ZnO Murray material (reproduced with permission [217], Copyright 2017, Macmillan Publishers Ltd.).

A variety of organisms in nature have naturally evolved into special forms with hierarchically porous architectures in order to achieve efficiency and exchange, in which the pore sizes are regularly organized across multiple scales [210,211]. By studying the mass transfer behavior of living organisms, a principle named as Murray's law has been proposed, which describes a condition under which an entire natural porous network connected within a finite volume can minimize the transport resistance of all pores and ensure fluent transfer throughout the whole network [212–215]. For example, in order to optimize the transfer and exchange of nutrients and water to enhance photosynthesis, the quantitative matching of stem diameters of a plant from macro to micro levels is strictly governed by Murray's law [216]. The applications of inorganic materials are often restricted by their low mass transfer efficiency. How to design and develop materials with high transport rate has always been a problem. Although Murray's law was discovered practically one hundred years ago to reveal efficient diffusion in nature, no one has yet used it to design advanced materials to maximize mass transfer for improving the performance and process efficiency of materials. By introducing the law of mass conservation into Murray's principle, our group [217] creatively deduced the generalized Murray's law involving mass variation: $r_0^\alpha = \frac{1}{{1 - X}}\ \mathop \sum \nolimits_{i\ = \ 1}^N r_i^\alpha $ (*r*_0_ is the radius of parent pore, *r_i_* is the radius of child pore, *N* is the numbers of pores, *X* is the ratio of mass variation during mass transfer, and the exponent *α* (2 or 3) is dependent on the types of the transfer). This law gives a strict mathematical relationship for the aperture matching of pore sizes between different scale levels. Following this generalized Murray principle and using a bottom-up approach, a novel hierarchical porous ZnO Murray material has been designed and synthesized (Fig. 13a and b). Their hierarchical macro-meso-microporosity as the system observed in natural vascularized materials is topographically highly interconnected, with the diameter of micropores, mesopores and macropores around ∼1 nm, ∼18 nm and ∼1000 nm, respectively (Fig. 13c–f). Because their pore sizes, ranging from micro to macro levels, fit the generalized Murray law, in the process that depends heavily on mass diffusion or ion transport the hierarchically porous ZnO Murray materials are considered as potential medium for various applications. Indeed, this kind of hierarchical ZnO Murray material with three-level porosity exhibits a high reversible capacity of 870 mAh g^−1^, even at the extremely high current density of 20 A g^−1^ (Fig. 13g). Moreover, excellent long-life cycling stability is also gained from these hierarchical ZnO Murray materials with a reversible capacity of 1260 mAh g^−1^ at a current density of 2.5 A g^−1^ over 5000 cycles (Fig. 13h), which is more than 25 times higher than that of state-of-the-art graphite. Although few new Murray materials have been reported, accurate design and quantitative matching of pore size for hierarchically structured porous materials will greatly improve their electrochemical properties. It can be predicted that this strategy can be applied to various porous materials and has broad prospects for energy, environment and medicine applications.

### Geometric configuration of the pores

The geometric configuration of pores is also a key factor that affects the properties of hierarchically structured porous materials. The geometric configuration of pores involves a wide range of areas, mainly including the shape of pores, the arrangement of pores and the orientation of pores, and so on. The shapes of the pores are various, the arrangement of the pores can be divided into ordered pores and disordered pores, and the orientation of the pores can be centrally divergent or have parallel/vertical alignment. In this part, since there is not enough research that can lead to a defined conclusion on which geometric configuration of hierarchically structured porous materials can perform better electrochemical properties, we will thus evaluate the electrochemical behavior of hierarchically structured porous materials with different geometries to provide guidance and also to draw more research attention for the structural design of hierarchically structured porous materials used in energy storage applications.

#### Shape of the pores

**Figure 14.**
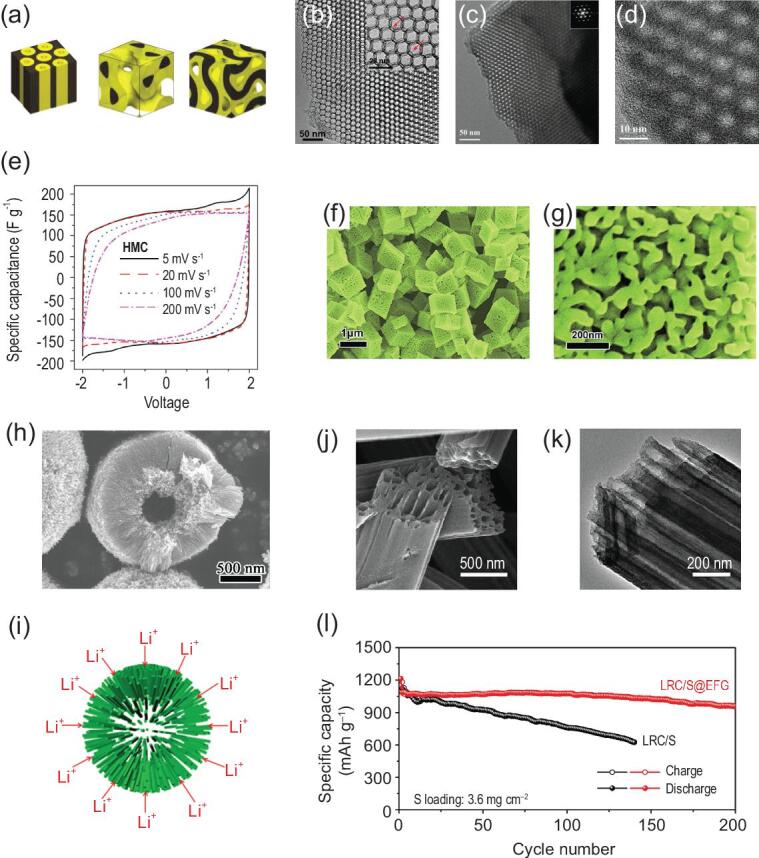
(a) Schematic diagram of carbon-sulfur composites from cylindrical, single gyroidal and double gyroidal porous carbon network (reproduced with permission [218], Copyright 2015, American Chemical Society); (b) TEM image of graphitic-carbon frameworks with cage-like mesoporosity (reproduced with permission [219], Copyright 2017, Springer); (c) TEM image, (d) high-resolution TEM images of hierarchically structured porous carbons with ordered mesoporosity and (e) CV curves of the fabricated carbon materials in neat ionic liquid EMIMBF_4_ electrolyte with a two-electrode system (reproduced with permission [159], Copyright 2016, Elsevier Ltd.); (f) low magnification SEM image and (g) high magnification SEM image of hierarchical porous Mn_2_O_3_ with disordered porosity (reproduced with permission [132], Copyright 2015, Macmillan Publishers Ltd.); (h) SEM image and (i) Li^+^ transport mechanism of hierarchically porous TiO_2_ hollow microspheres with radially oriented nanochannels (reproduced with permission [222], Copyright 2015, Elsevier Ltd.); (j) SEM image and (k) TEM image of the hierarchical porous carbon nanofibers with parallel channels, and (l) cycling performance of the fabricated carbon nanofibers (reproduced with permission [224], Copyright 2015, Macmillan Publishers Ltd.).

In the field of energy storage, the structural characteristics and the shapes of pores have an important influence on the performance of hierarchically structured porous materials. To understand these correlations, precise and smart synthetic control over the morphology of the pores is urgently required. Through a templating method with simply changing the ratios of copolymers to precursors, Werner *et al.* [218] synthesized a series of hierarchically micro/mesoporous carbon materials with single gyroidal mesopores, double gyroidal mesopores and hexagonally packed cylindrical mesopores, respectively (Fig. 14a). At a charge-discharge rate of 0.1 C, micro/mesoporous carbon hosts for sulfur with the above three structures were tested to evaluate their ability to trap polysulfides. Despite obvious differences in pore shape and volume between the hexagonally packed cylindrical carbon and the gyroidal carbons, all carbon hosts show very similar and poor performance over cycling with a relatively low initial capacity and rapid capacity fade. This is mainly due to the fact that these three kinds of carbon hosts, although they have different pore shapes, contain open pore channels, which is not conducive to restricting the shuttle effect of polysulfides. Therefore, some hierarchically structured porous materials with specific closed or partially closed pore shapes could be advantageous, such as funnel-like pores, cage-like pores and bottleneck pores [219,220]. For example, Yu *et al.* prepared porous graphitic-carbon frameworks (MGFs) with hierarchical micro-mesoporosity derived from ordered superlattices by the self-assembly of Fe_3_O_4_ nanoparticles, which have half-closed cage-like mesoporous architectures interconnected by microporous openings on their pore walls [219] (Fig. 14b). With special pore shape and advantageous structural features, the cage-like mesoporous ‘houses’ efficiently trap sulfur and suppress the diffusion of polysulfides while the microporous ‘windows’ ensure rapid electron and lithium-ion transport. Thus the as-fabricated S@MGFs show excellent electrochemical properties in discharge capacity, rate performance and cycle stability for Li-S batteries. The structure also demonstrates good performance for LIBs owing to the opened pore shape. By template-assisted one-pot spray pyrolysis, Ko *et al.* [221] prepared MoO_2_-C porous microballs with ant-cave-shaped macroporosity. Macroporous channels this shape are well interconnected and provide plenty of accessible pathways, facilitating the penetration of electrolyte and allowing an efficient electron/ion transport. As anode for LIBs, it thus exhibits superior electrochemical performances.

The shape of the pores not only affects the performance of the electrode material, but also can provide ideas for the design of the pore structure of the separator in the batteries. At present, the modification of separators is mainly by surface coating methods. We believe that the performance of lithium-sulfur batteries can be greatly improved by preparing separators with wedge-shaped or funnel-shaped pores based on the design of the pore structure; because the pore sizes of the two sides of the separators are not the same, the side with the smaller pore size can effectively inhibit the polysulfide transport to the lithium anode without affecting the normal transport of lithium ions.

#### Arrangement of the pores

The arrangement of the pores can be divided into ordered pores and disordered pores. These two arrangement forms are both useful in energy applications. Recently, a block copolymers-assisted self-assembly method has been reported to synthesize hierarchically micro-mesoporous carbons, in which the hybrid molecule of polyhedral oligosilsesquioxanes (POSS) uses as self-template and the triblock copolymers of PEO-PPO-PEO act as soft templates [159]. The obtained carbon materials have uniform micropores and highly ordered mesopores (Fig. 14c and d). Their highly ordered mesoporous structure facilitates the rapid transport of ions into tiny micropores, resulting in superior power properties. It is confirmed by experimental data that the hierarchically porous carbon sample with an ordered hexagonal mesostructure displays a high rate capability with 94% of capacitance retention in ionic liquid electrolyte when the current density changes from 0.25 to 10 A g^−1^ (Fig. 14e). 3D hierarchically bimodal mesoporous Mn_2_O_3_ single crystals (Mn_2_O_3_ SCs) with highly interconnected porosity via the thermal decomposition of MnCO_3_ precursors have been successfully synthesized [132]. Although the arrangement of mesopores is disordered (Fig. 14f and g), they exhibit a bicontinuous and interconnected framework, thus also resulting in enhanced electrochemical properties for LIBs because this architecture can offer abundant active sites for the lithiation/delithiation reaction and facilitate lithium ion diffusion.

#### Orientation of the pores

The orientation of the pores determines the path of mass transport and electrons/ions diffusion. The radially oriented pore channels can effectively facilitate ions and electrolyte molecular transport from the surface of the material to the interior, which has a good prospect in the field of energy storage. The hierarchically porous TiO_2_ hollow microspheres were one-pot facilely prepared by a fluorine-free solvothermal method [222]. The shells of the hollow spheres are formed by the radial assembly of nanorod chains, resulting in a large series of straight centrally divergent channels along nanorod chains (Fig. 14h). Such straight radially oriented nanochannels are highly desirable for lithium ion batteries because such orientation of the pores can facilitate the diffusion of electron/ion and buffer the volume change during the change/discharge process (Fig. 14i). The obtained TiO_2_ anode materials with such orientation of the pores demonstrates excellent Li^+^ storage capacity with exceptional cycle performance (a high reversible capacity of 216 mAh g^−1^ after 100 cycles at 1 C) and superior rate capability (a reversible capacity of 112 mAh g^−1^ after 100 cycles at 10 C). In addition, the hierarchically meso-macroporous carbon materials with parallel macrochannels were prepared [223]. Such channel-oriented structure is very suitable for separation and purification processes. Learning from this, parallel-oriented pore structures also have potential advantage in energy storage applications. Li *et al.* designed and developed a ‘pie’ structured electrode constructed by 3D interconnected lotus root-like multichannel carbon (LRC) nanofibers [224]; there are many highly parallel channels in each nanofiber (Fig. 14j and k). Compared to disordered porous structures, the highly parallel channels form excellent conductive skeletons at the nanoscale, providing effective electron/ion accessibility. Each channel within the LRC nanofibers acts as a nanoscale electrochemical reaction chamber, resulting in a more complete redox of the active materials, due to the close contact between restricted sulfur and intermediate lithium sulfide, delivering a high specific capacity of 1314 mAh g^−1^ (equivalent to areal capacity of 4.7 mAh cm^−2^) at 0.1 C (equivalent to areal current density of 0.6 mA cm^−2^) as well as excellent cycling stability (Fig. 14l).

In this section, the effect of organizational, structural and geometric parameters of a pore in hierarchically structured porous materials on their energy storage performance has been discussed and is clearly evidenced. For the sake of clarity, a large series of hierarchically structured porous materials with different porous hierarchy, different pore parameters and different chemical compositions are compared in Table 2 in energy storage application. Their performance and preparation methods are also given. This table can give a first guide to readers when selecting materials for a specific energy storage application.

## SUMMARY AND PROSPECTS

The progress and successes in synthesis of hierarchically structured porous materials have rendered it possible to tune their diffusion behavior and chemical kinetics. These superiorities in turn have stimulated the exploitation of various energy storage materials and devices. With the development of economy and society, high-performance energy storage materials and devices with high energy and power densities, long lifetime and excellent stability are significantly required. As a kind of biomimetic material, the hierarchically structured porous materials possess inherent structural advantages that follow the laws of nature, showing extraordinary potential in the field of energy storage. In particular, by adjusting and designing the parameters of pores, such as number of the scale levels, size of the pores and geometric configuration of the pores, the transfer efficiency of materials and ions/electrons can be maximized, and the electrochemical properties of the materials can be brought into the optimal status. Synergy effect from hierarchical porosity across multi-length scales, quantitative matching of pore sizes ranging from micro- to macro-levels and the fine control of the geometric configuration of the pores, have proven to be effective and versatile strategies for designing advanced electrode materials. With this in mind, it is crucial to accurately tune the pore parameters by the use of multidisciplinary knowledge from material science, chemistry, physics and biology for energy storage applications.

Despite substantial progress in the development of hierarchically structured porous materials for electrochemical energy storage, some critical drawbacks still exist and impede their commercial applications. Generally, hierarchically porous structures endow materials with high surface area and fast mass transport. This however increases the chance of side reactions in another way. In particular, these undesired side reactions will lead to the consumption of electrolyte, irreversible capacity loss and the decrease of the utilization of active materials, which is detrimental to the battery properties. The other obstacle is the low volumetric energy density due to the high porosity of hierarchically structured porous materials. Integration and miniaturization are the trend of electronic products, which heavily depends on novel electrode materials with high volume energy density. This poses a great challenge to the hierarchical porous materials for energy storage application in the near future.

Solutions to the challenges faced by hierarchically structured porous materials require new discoveries and new thoughts, which render stimulating opportunities for those entering this field. Functional modification of pore channels to reduce the occurrence of side reactions, controlling pore crystallinity to improve thermal stability and quantitative matching of pore sizes across multiple length scales to optimize mass transfer, are some exciting and intriguing research directions in the future. With the knowledge on hand on the relationship between the organizational, structural and geometric parameters of pores at different length scales and the electrochemical performance drawn from this review, it is possible to establish a series of material design rules to synthesize hierarchically structured porous materials via different synthesis technologies presented in this review with predictive and optimized properties from porous hierarchy for high performance energy storage applications.
